# Discovery of Influenza Neuraminidase Inhibitors: Structure-Based Virtual Screening and Biological Evaluation of Novel Chemotypes

**DOI:** 10.3390/molecules30234636

**Published:** 2025-12-02

**Authors:** Rosaria Gitto, Lisa Lombardo, Angela Ravenda, Francesco Broccolo, Antonio Mastino, Laura De Luca, Francesca Marino-Merlo

**Affiliations:** 1 CHIBIOFARAM Department, University of Messina, Viale F. d’Alcontres 31, I-98166 Messina, Italy; lisa.lombardo@studenti.unime.it (L.L.); laura.deluca@unime.it (L.D.L.);; 2 DiMeS Department of Experimental Medicine, University of Salento, Ecotekne Center, S.P6 Monteroni, I-73047 Lecce, Italy; francesco.broccolo@unisalento.it; 3 The Institute of Translational Pharmacology, CNR, Via Fosso del Cavaliere 100, I-00133 Roma, Italy; antonio.mastino@ift.cnr.it

**Keywords:** neuraminidase, anti-flu agents, NAIs, MD simulation, pharmacophore modeling, flexible docking, virtual screening

## Abstract

Neuraminidase (NA) decorates the surface of the influenza virus, exerting a sialidase activity that enables the viral particle to be released in the host cell. Numerous sialic-based antiviral agents competitively bind to the NA cavity and are marketed worldwide for the treatment of Influenza A infection. We designed and validated a structure-based pharmacophore model for influenza neuraminidase (NA), which guided a virtual screening campaign against an in-house library of compounds already available for testing. This fast and cost-effective in silico strategy resulted in the identification of seven candidates possessing indole or isoquinoline chemical core. In vitro assays confirmed their favorable cytotoxicity profiles and identified only one, the 1-(1*H*-indol-3-ylcarbonyl)-3-piperidinecarboxylic acid (1), with reproducible inhibitory activity toward NA at non-cytotoxic concentrations. This work suggested a validated workflow for the discovery of novel NA inhibitors and highlighted an indole-based hit compound as a starting point for further optimization.

## 1. Introduction

**Figure 1:**
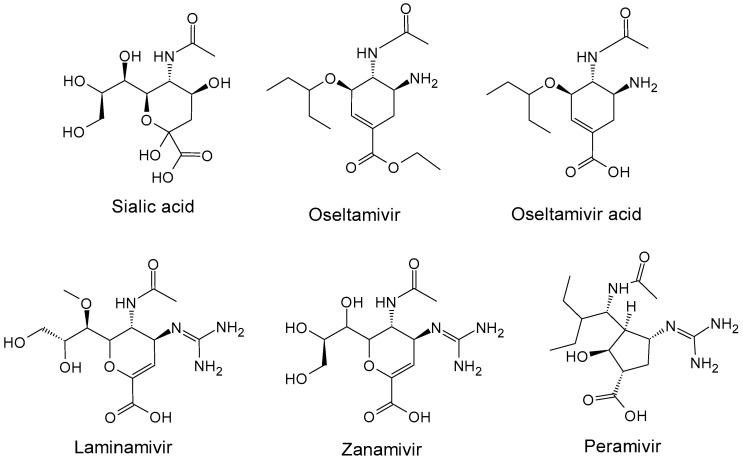
Sialic acid (SA) and SA-inspired compounds acting as neuraminidase (NA) inhibitors.

Influenza viruses represent a concern for public health by causing contagious seasonal epidemics such as respiratory diseases in humans; additionally, spillover events facilitate zoonotic transmission and heighten the risk of interspecies adaptation. The influenza viruses belong to the *Orthomyxoviridae* family and contain single-stranded negative-sense RNA genomes. There are four distinct antigenic types of influenza viruses (type A, B, C and D); among them influenza A and B viruses (IAV, IBV) induce mild to severe respiratory illness in humans almost every winter (seasonal epidemics) [1]. The IAV subtypes are named on the basis of a pair of glycoproteins named hemagglutinin (HA) and neuraminidase (NA) that decorate the surface of the viral particles. Moreover, IAVs undergo genetic variations (also known as antigenic shift) during pandemic events in different hosts including humans, pigs, horses, birds, reptiles, and so on. Annual vaccination provides valuable protection against seasonal influenza and remains the most effective preventive strategy against influenza-associated morbidity and mortality [2,3,4,5,6]. However, seasonal vaccines could reduce their efficacy by year, population under study, and virus strains. The IAV replication cycle comprises distinct steps [7,8] including the processes of anchoring to epithelial cell, endocytosis, uncoating and membrane fusion, and subsequently replication by the RNA polymerase; finally, the new viral particles are released for dissemination to other cells. HA and NA proteins promote the anchoring of IAV on the host cell surface as well as the virus spreading through the budding of newly emerged virions from the host cells to neighboring healthy cells. Particularly, NA catalyzes the hydrolysis of terminal sialic acid (SA, Figure 1) from sialyloligosaccharides.

To date, anti-influenza (anti-flu) agents comprise therapeutics which generally are virus-directed agents targeting different steps of IAV replication cycle [8,9,10,11,12,13] as NA inhibitors (NAIs), ion channel blockers to interact the viral ion channel M2, viral polymerase inhibitors addressing their activity towards PA, PB1, and PB2 subunits, and HA inhibitors able to impair membrane fusion processes. Up to now, NA was considered the most successful drug target, to the point where several SA analogues (Figure 1) [14,15,16,17] such as oseltamivir, zanamivir, and peramivir were approved by FDA and EMA; in addition, laninamivir is currently in clinical investigation and is approved only in Japan. Unfortunately, the IAV ability to continuously evolve in mutant strains [18] challenges the design of newer effective anti-flu agents. Actually, the mutations that occur in new strains, in addition to eluding the pre-existing natural or vaccination-based immune response, can also limit the currently employed inhibitors as well by eluding the pre-existing immune response also based on anti-flu vaccination. Given the considerable effects of anti-flu agents acting as NA inhibitors, many efforts focused their attention on the development of further SA analogues with improved safety and tolerability as well as efficacy toward resistant strains [19,20,21,22]; therefore, various oseltamivir-inspired compounds are undergoing clinical trials [23].

It is well-known that IAVs possess distinct variants based on the presence of different HA (1–18) and NA (1–11) protein types. From a structural point of view, the NA is a homotetrameric type II transmembrane protein and nine subtypes from N1 to N9 were identified in humans; they were classified into two distinct groups according to their primary sequences [24,25]. In detail, group 1 comprises N1, N4, N5, and N8; whereas, group 2 comprises N2, N3, N6, N7, and N9; additionally, only group 1 displays the so called “150-cavity” adjacent to the active site generated by the movement of 150-loop (residues 147–152) during the binding of SA within the sialidase active site. Unlike most group 1 NAs, the NA of the 2009 pandemic H1N1 influenza virus (09N1) lacks a 150-cavity in its active site, making it an atypical member of this group [26].

The NA is arranged in a square disposition, for which the tetrameric architecture is optimal for enzyme activity. Each monomer is composed of four structural domains: (a) the catalytic head, (b) the stalk, (c) the transmembrane region, and (d) a highly conserved C-terminal cytoplasmic tail. The NA catalytic head consists of a box-shaped structure comprising four monomers; the functional catalytic site is present on the surface of each monomer, forming a large cavity possessing a large number of charged residues. The crystal structures of the box-shaped NA head domain have been determined for all IAV NA subtypes [27] revealing that the active site is generally highly conserved making it an ideal target for drug inhibition since it contains information for structure-based design of antiviral agents [28]. Moreover, the stalk domain possesses cysteine residues assisting tetrameric stabilization by enabling disulfide bonds; moreover, the presence of carbohydrate sides further contributes to the stability of the tetrameric NA [25]. It has been demonstrated that the stalk length regulates the virulence in mammalians [25].

The hydrophobic N-terminal transmembrane domain links the protein to the viral envelope and provides signals for translocation of the apical surface. It has been established that the well-known NA inhibitors oseltamivir and zanamivir mimic the binding mode of the transition state analogue 2-deoxy-2,3-dehydro-N-acetylneuraminic acid (DANA) as natural substrate within the catalytic site with eight crucial residues R118, D151, R152, R224, E276, R292, R371, and Y406 [25,29]. The reduction of sensitivity to NA inhibitors can be conferred by the mutation of several residues located within or close to the active site (E119, I122, Q136, D151, R152, D198, R224, S246, H275, R292, N294, and R371) [18]; some of them do not directly establish contact with SA but possess a structural role for NA such as D198 and N294; the most frequent mutation regards the H275Y substitution (N1 numbering) generating resistance to NAIs such as oseltamivir and peramivir. Moreover, the mutation of specific amino acids within the transmembrane domain induces differences in the anchoring signal region and reduced transport to the plasma membrane [30].

**Figure 2:**
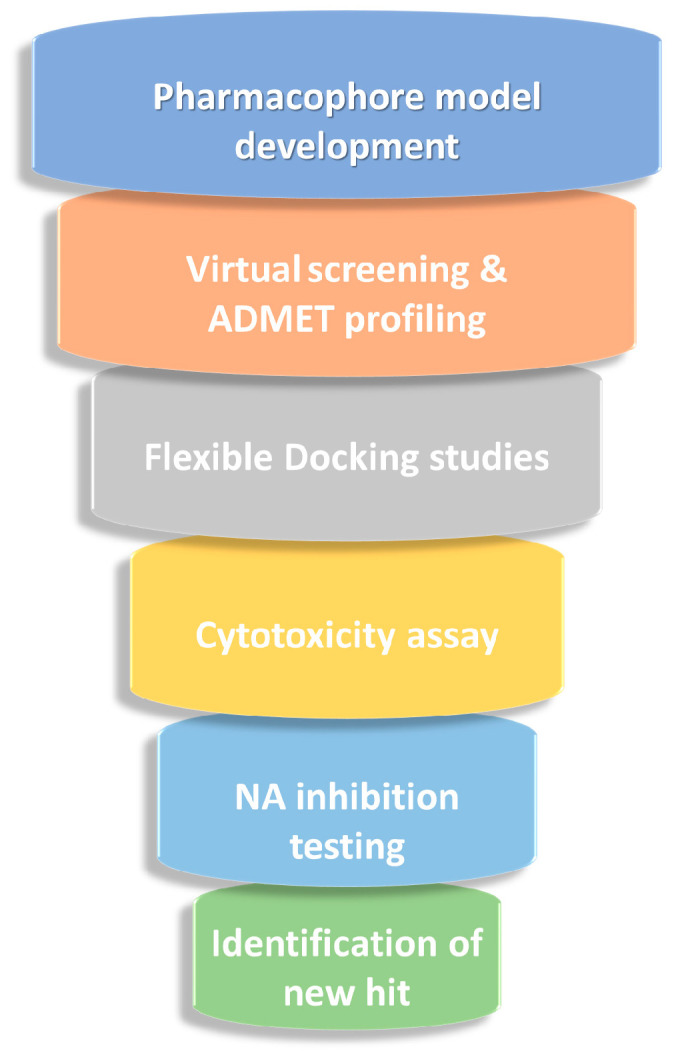
General workflow of our computational study for identifying NAIs.

Extensive efforts have been made to overcome the poor pharmacokinetic and resistance issues associated with competitive NA inhibitors as SA-analogs through structural modifications designed to enhance lipophilicity, selectivity, and binding to the catalytic pocket, including adjacent residues of the 150- and 430-loops (residues 430–439). However, the continuous emergence of resistant viral strains underscores the urgent need to expand the structural diversity of NAIs and explore new chemotypes beyond well-known scaffolds. Various researchers were engaged in development of new NAIs harnessing natural-inspired small molecules as well as heterocyclic-based compounds from synthetic sources [22,31,32,33]. In this framework, our study was conceived to identify innovative small-molecule candidates with a chemical core distinct from oseltamivir and related analogs, while preserving the ability to interact with the catalytic site of the pandemic H1N1 neuraminidase (09N1). To achieve this objective, we implemented a multi-step computational strategy summarized in Figure 2, integrating computational modeling, virtual screening, and preliminary biological validation.

In more detail, we selected the 2015 variant of 09N1 as our protein template, introducing its reported mutations into the available 2009 crystal structures without affecting active-site residues. On this basis, we developed a structure-based pharmacophore by aligning and comparing three ligand-bound N1 crystal structures in complex with known inhibitors. Subsequently, a structure-based virtual screening (VS) and ADMET prediction were conducted within our in-house database containing a large set of heterocyclic compounds already synthesized and tested for different therapeutic targets. Then, we carried out computational docking analyses to unveil the binding mode within the NA catalytic pocket. In the final step, the top-ranked selected molecules were experimentally tested for cytotoxicity and neuraminidase inhibition using recombinant H1N1 enzyme and the fluorometric MUNANA assay.

## 2. Results

### 2.1. Computational Studies on Neuraminidase

#### 2.1.1. Pharmacophore Model Development and Virtual Screening

To capture the three-dimensional organization of NA and highlight the crucial chemical features and key residues controlling binding interaction of NAIs, we built a merged structure-based pharmacophore model to guide the identification of new NAIs. The merged model was from three crystal structures of NA from the influenza A (H1N1) virus, strain A/California/04/2009, bound to laninamivir, zanamivir, and oseltamivir acid (PDB IDs: 3TI3, 3TI5, and 3TI6) [34]. We selected the crystal structures of the pandemic A/California/04/2009 (H1N1) neuraminidase that represented the highest-resolution templates available for group 1 NAs; then, we decided to compare them with a more recent virus strain A/Michigan/45/2015 (N1) in apo state (PDB ID: 7S0I), also belonging to pdm09-era N1 (lacking the 150-cavity) and presenting several mutations when compared to the 2009 strain [35]. Sequence analyses indicated that A/Michigan/45/2015 and A/California/04/2009 neuraminidases shared more than 98% sequence identity, differing by only ten residues located mainly on the outer surface of the head domain rather than within the catalytic pocket [36]. Therefore, in silico mutagenesis was performed to introduce variant residues of the N1 2015 strain into the three selected complex structures (PDB IDs: 3TI3, 3TI5, and 3TI6) of the N1 2009 strain, as described in Section 3 for the following residues N199S, V240I, N247D, V263I, N269K, I314M, I321V, N372K, N389K, and K432E (430-region) that, in not belonging to the catalytic area, were therefore not expected to influence ligand binding or enzymatic inhibition under our experimental conditions. The above-mentioned approach was consistent with in silico mutagenesis strategies that have been successfully employed in previous neuraminidase modeling studies, where closely related subtypes were refined by introducing strain-specific mutations to reproduce the active-site environment of contemporary isolates [37,38].

In turn, the mutated complexes underwent a multi-step energy minimization protocol as detailed below. The mutated residues and all atoms within a 2 Å radius were initially minimized using MacroModel [39] and Desmond [40] tools of the Schrödinger Suite. Subsequently, the refined complexes were submitted to 100 ns molecular dynamics (MDs) simulations to assess their conformational stability and ligand–protein interactions. All complexes demonstrated stable Root Mean Square Deviation (RMSD) profiles with persistent ligand–target contacts that were maintained throughout the simulations (Figures S1 and S2 in Supplementary Materials). This hybrid approach enabled us to capture the subtle conformational adjustments observed in post-pandemic N1 variants while preserving the experimentally validated structural framework of the 2009 enzyme.

**Figure 3:**
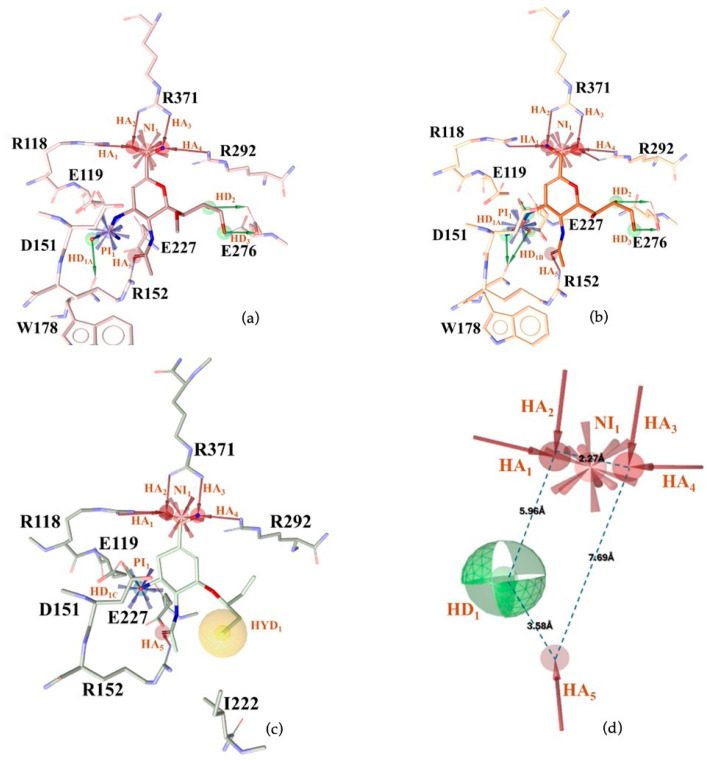
Structure-based pharmacophore models generated from N1 bound to (**a**) laninamivir (in pink), (**b**) zanamivir (in orange), and (**c**) oseltamivir acid (in pale green). Both residues and ligands are depicted as sticks. (**d**) Final pharmacophore model obtained by our consensus approach. Features are shown as follows: hydrogen bond acceptors (HBAs) and hydrogen bond donors (HBDs) as red and green arrows, respectively, hydrophobic areas (HYDs) as yellow spheres, positive (PI) and negatively ionizable (NI) regions as blue and red stars, respectively, and higher tolerance hydrogen bond donor featured as green sphere. Excluded volumes are not displayed for clarity.

Using LigandScout v4.5 [41] we created the corresponding three structure-based pharmacophore models depicted in Figure 3a–c for N1 bound to laninamivir (pink), zanamivir (orange), and oseltamivir (pale green) within the cavity devoted to substrate SA.

Overall, the visual inspection of the structure-based pharmacophore models for the three inhibitors confirmed that they exhibited a very similar binding mode and formed crucial contacts with key polar residues R118, E119, D151, R152, W178, E227, E276, R292, and R371 characterizing both the 2009 and 2015 N1 strains. In detail, the pyran ring of zanamivir and laninamivir and the cyclohexene system of oseltamivir acid shared a similar orientation in the active site. Additionally, the functional groups decorating the core contributed to analogous interactions. Notably, for all compounds, the carboxylate group established hydrogen bonds and ionic interactions with the arginine triad R118, R292, and R371, contributing to establishing five polar contacts that combined HA (HA_1–4_) and NI_1_ features. The common acetamido group acting as the HA (HA_5_) feature forming a hydrogen bond interaction with R152 for all studied inhibitors. As shown in Figure 3, the cationic moieties formed both hydrogen bonding and electrostatic interactions as found for the primary amine of oseltamivir and the guanidino groups both of zanamivir and laninamivir; these functionalities served as HD (HD1_A–C_) and PI_1_ features engaging profitable contacts with carboxylate of E119, D151, and E227, as well as the oxygen atom of W178 backbone. As expected, the presence of distinct functional groups linked to the C-6 position of the core scaffold led to differences in several pharmacophoric features. In more detail, zanamivir and laninamivir contained a glycerol-like moiety (2,3-dihydroxy-1-methoxypropyl chain for laninamivir and 2,3-dihydroxypropyl chain for zanamivir), which interacted with E276 residue, contributing to two HBD features (HD_2_ and HD_3_) (Figure 3a,b). In contrast, oseltamivir possessed a bulky ethylpropoxyl group, which induced a conformational shift of the E276 side chain due to steric hindrance, favoring hydrophobic interactions (HYD_1_) with I222 (Figure 3c) in place of polar contact with E276 residue.

To develop a more restrictive screening tool for novel NAIs, we constructed a shared pharmacophore by merging the three models shown in Figure 3a–c. Through alignment-based integration, we retained only the common interactions characterizing all three target–ligand complexes; as a result, we obtained a highly simplified yet robust pharmacophore model (Figure 3d). This shared pharmacophoric hypothesis comprised four hydrogen bond acceptors (HA_1–4_) and one negative ionizable feature (NI_1_) from carboxylate interactions with R118, R292, and R371, plus one acceptor (HA_5_) from acetamide–R152 binding. To accommodate the structural flexibility, the hydrogen bond donor formed between oseltamivir and E119, or between laninamivir/zanamivir and W178, was represented as a spherical feature with increased tolerance (HD_1_). The PI_1_ region common to all three models was merged into HD_1_ to account for potential protonation of the donor moiety. Moreover, eight excluded volumes were maintained to improve virtual screening effectiveness.

To test the discriminatory power of this simplified pharmacophore model, we performed a virtual screening procedure on a custom-designed validation set. The compound library was retrieved from the BindingDB database (https://www.bindingdb.org/, accessed on 15 March 2025) including only molecules with experimentally measured IC_50_ values against the H1N1 influenza strain. As detailed in Section 3, the dataset was curated by removing duplicates and outliers. Subsequently, selected molecules were classified into two subsets: an active set (IC_50_ < 10 nM), consisting of 87 compounds, and an inactive set (IC_50_ > 10,000 nM), comprising 507 compounds.

**Figure 4:**
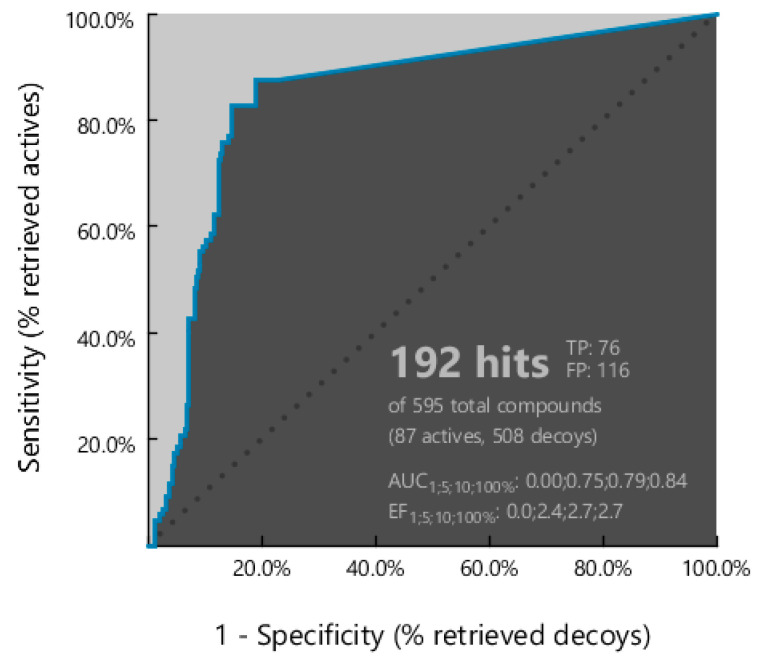
ROC-AUC obtained by the validation procedure, along with the corresponding AUC and EF output values.

Additionally, pharmacophore performance was assessed using standard metrics implemented in LigandScout [41], including the receiver operating characteristic (ROC) curve, area under the ROC curve (AUC), and enrichment factor (EF) (Figure 4). The model correctly identified all 87 active compounds (true positives) and misclassified 116 inactive compounds as active compounds (false positives). The resulting AUC of 0.84 reflects excellent discrimination between active and inactive molecules. Furthermore, the enrichment factor (EF) of 2.7, calculated over the full dataset, indicated a moderate enrichment capacity, confirming that the pharmacophore model significantly outperformed random selection. Taken together, the strong AUC and reasonable EF values supported the robustness and predictive utility of the model.

To discover new NAIs we applied the validated pharmacophore model to virtually screen our proprietary library of 1683 compounds (CHIME2024), which have been already synthesized and/or characterized for biological targets other than NA. Specifically, our repository contained small molecules distinct from canonical sialic-based compounds but featuring the carboxylic or sulfonamide group as key moiety for anchoring to the NA cavity.

Given the limited dataset size, a flexible pharmacophore screening approach was adopted, allowing the omission of one feature per compound. This strategy was intended to maximize hit retrieval while preserving the relevance of the interaction profile. The virtual screening campaign yielded 298 hits. Subsequent filtering steps were applied to reduce the number of hits by considering chemical stability, compliance with Lipinski and lead-like rules, and aqueous solubility, with these parameters evaluated using the Percepta tool [42] of ACD/Labs software (version 2025); by applying these filters, we selected a collection of 165 molecules meeting the established selection criteria.

Finally, the compound selection was carried out by experience-based visual inspection of binding modes for which the priority was made based on the matching of all pharmacophore features or considering admissible only the lack of either an H_5_, N_1_, or HD_1_ feature. Table 1 collects the seven selected candidates **1**–**7** and their pharmacophore matching data, mapping, and fit scores. The virtual screening filtered only the (*S*)-stereoisomer for compounds possessing a chiral center. In more detail, indoles **1**–**3** matched all chemical features, indole derivative **4** missed the HA_5_ feature, isoquinoline **5** lacked the HD_1_ feature, and isoquinolines **6**–**7** were missing the NI_1_ feature. From a chemical point of view, compounds **1**–**3** shared the indole-3-carbonyl core connected to a terminal carboxylate through a distinct linking portion: a conformationally constrained azacyclic amide in **1**, a flexible linear amide in **2**, and the related isosteric ketone linker in **3**. In contrast, compound **4** was characterized by the indole ring decorated with the carboxylate functionality, thus lacking the amide/ketone functionality as an H_5_ pharmacophoric feature. Compound **5** retained the carboxylate group but lacked the HD_1_ feature, while compounds **6** and **7** did not possess the N_1_ feature and were lacking carboxylate substituents that were replaced by a sulfonamide moiety.

#### 2.1.2. Flexible Docking Studies

To further investigate the ability of selected compounds **1**–**7** to occupy the catalytic pocket of N1 of the crystal structure of N1 (PDB 3TI5), a flexible docking procedure was performed using the GOLD program [43]; then we applied the rescoring of the docking poses as described in Section 3. We validated our docking protocol through a comparative analysis of three PDB complexes (see Supplementary Materials Figure S4A–C). The three catalytic sites displayed high structural conservation with minor variations at positions S246, D247/N247, E276, and N347. Structural superposition yielded protein RMSD values below 1.3 Å, and re-docking experiments produced ligand RMSDs below 1.1 Å. Based on these results, 3TI5 was selected as the reference structure for all subsequent docking calculations.

**Figure 5:**
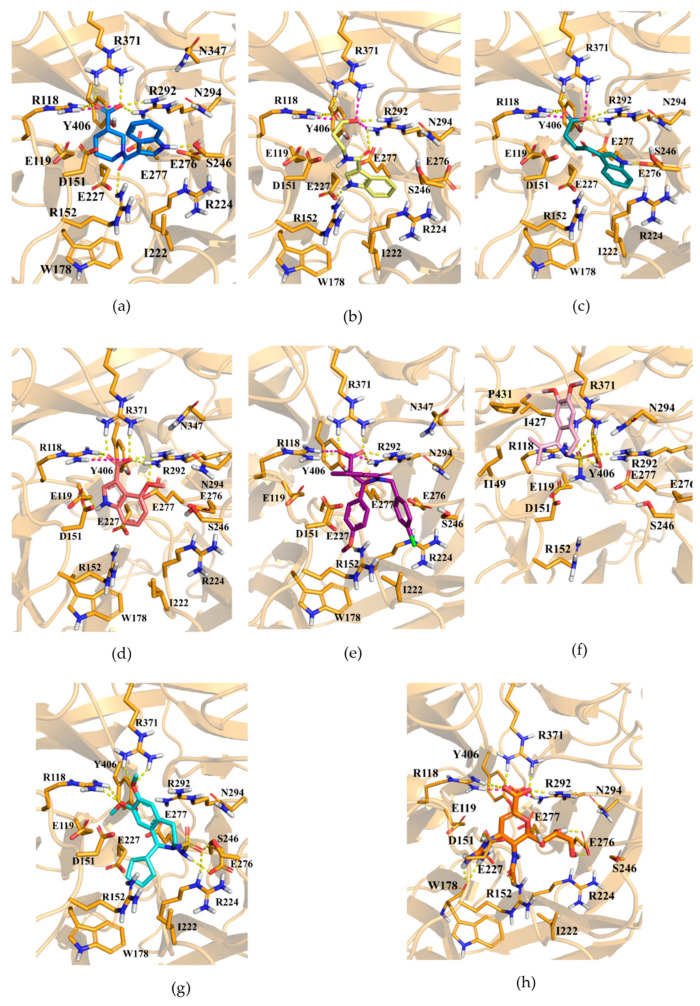
Docking poses of compounds (**a**) **1** (in marine blue sticks), (**b**) **2** (in yellow sticks), (**c**) **3** (in deep teal sticks), (**d**) **4** (in salmon sticks), (**e**) **5** (in purple sticks), (**f**) **6** (in pink sticks), (**g**) **7** (in cyan sticks), and (**h**) the X-ray structure of zanamivir (in orange sticks, PDB 3TI5) in the binding site of N1. Residues of the catalytic site are shown as bright orange sticks, while the remaining part of the protein is displayed as cartoon. Hydrogen bond interactions and ionic contacts are depicted as yellow and magenta dashes, respectively.

Compounds **1**–**7** were docked into the catalytic pocket of N1, and the resulting complexes are shown in Figure 5a–g, alongside the X-ray structure of zanamivir bound to N1 (PDB 3TI5, Figure 5h). All compounds occupied the active site similarly to zanamivir. Docking analysis revealed that derivatives **1**–**7** presented a low ASP score and a different ranking with respect to the pharmacophore fit score; however, all compounds were able to engage the catalytic tri-arginine cluster (R118, R292, R371) that was considered relevant for the sialidase activity of N1. Furthermore, there were hydrogen bonds involved, R152, E227, E277, Y406, and W178, consistent with the co-crystallized ligand. In this context, our indole-3-carbonyl amide/ketone derivatives (**1**–**4**) represented a novel synthetic implementation of this pharmacophore, with binding modes compatible with approved NAIs while preserving the key tri-arginine contacts [44,45,46]. In parallel, the isoquinoline core, previously described in natural alkaloids such as berberine and palmatine with NA inhibitory activity [47,48], was also retrieved in our library (compounds **5**–**7**), offering a complementary framework for exploration. Notably, hydrophobic interactions with I149, I427, and P431 were observed for compound **6** as well as for compounds **5** and **7** with residue I222. Such residues placed along the pocket rim/adjacent 150-/430-region were implicated in stabilizing NA–ligand binding, shaping 150-loop dynamics, and modulating NAIs susceptibility, proving to be important for inhibition activity [49,50,51,52]. Based on the results of VS, we decided to investigate the biological properties of selected compounds **1**–**7**. All derivatives were available in-stock in ~50 mg amounts in our repository CHIME2024 so there was no need to re-synthesize them or purchase from commercial suppliers. Before performing biochemical assay, compounds **1**–**7** were analyzed to ascertain their purity and chemical stability; as a result, the spectral data and chemical properties were in agreement with the proposed chemical structures (see Figures S5–S13 in Supplementary Materials); for all selected compounds **1**–**7**, we analyzed their most common physicochemical properties and their drug-likeness (see Experimental Section and Table S1 in Supplementary Materials).

### 2.2. Biological Evaluation

#### 2.2.1. Cytotoxicity Evaluation

**Figure 6:**
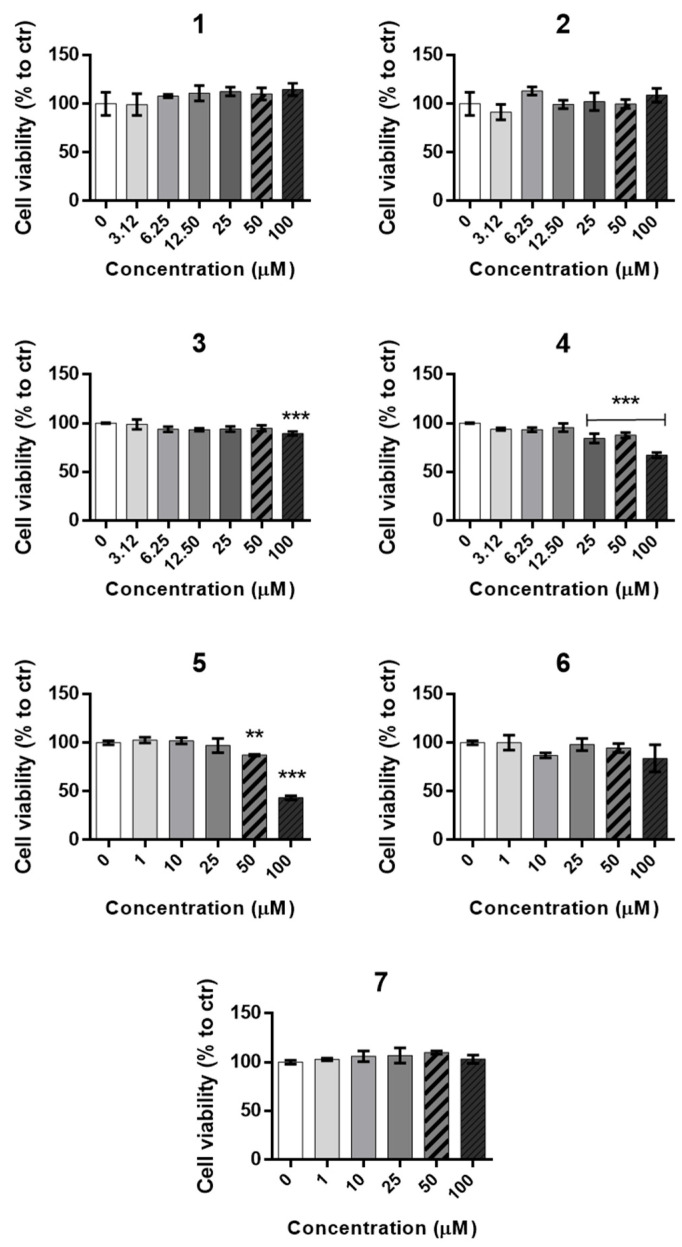
Cell viability assessed by metabolic activity (MTS assay) in HEp-2 epithelial cells treated for 24 h with the potential NA-targeting inhibitors. Each subfigure label corresponds to one of the seven tested compounds **1**–**7** at the indicated concentrations. Data are expressed as mean ± SD (n = 3 independent experiments performed in duplicate) and are presented as percentages relative to untreated control cells (0 µM), which were assigned a value of 100% cell viability. Statistical analysis was performed using one-way ANOVA followed by Bonferroni’s multiple-comparison test (** *p* < 0.01, *** *p* < 0.001 vs. control).

To define the safety window for biological testing of the potential antiviral activity of the compounds, cytotoxicity of the selected neuraminidase-targeting compounds was evaluated in HEp-2 epithelial cells by measuring the inhibition of cellular metabolic activity through the MTS viability assay. Cells were exposed for 24 h to serial concentrations (1–100 µM) of each compound, with DMSO (≤0.5%) as vehicle control (Figure 6). Cytotoxicity was expressed as the compound concentration reducing metabolic activity by 50% (CC_50_) compared to the corresponding vehicle-treated control.

Among the seven tested molecules, compounds **1**, **2**, and **7** showed no detectable cytotoxicity up to 100 µM, while compounds **3**, **4**, **5**, and **6** displayed CC_50_ values ranging from 152 µM to 670.6 µM (Table 2). Overall, these findings confirmed a favorable cytotoxicity profile for all tested molecules, with CC_50_ values well above the screening concentration, thus supporting the inclusion of 100 µM as the reference dose for the functional neuraminidase inhibition assays.

#### 2.2.2. Screening for Neuraminidase Inhibition

**Figure 7:**
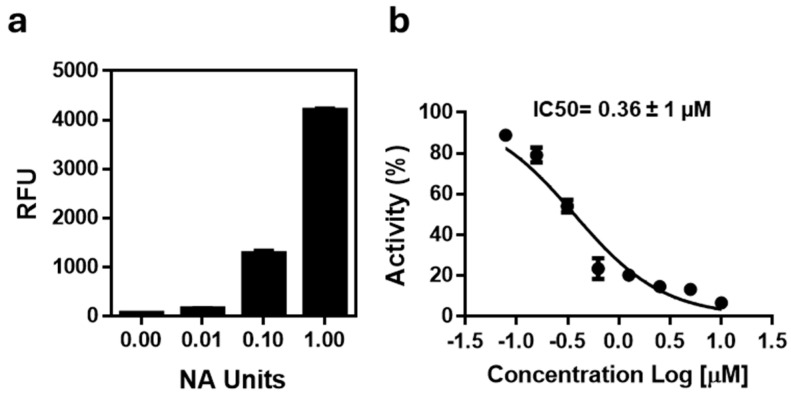
(**a**) Titration of recombinant H1N1 neuraminidase tested in the range of 0.01–1.0 U. (**b**) Dose–response inhibition curve of recombinant H1N1 neuraminidase treated with increasing concentrations of oseltamivir (0.08–10 µM). Data are mean ± SD from three independent experiments performed in duplicate.

To assess the ability of the selected compounds to inhibit neuraminidase (NA) enzymatic activity, a fluorometric assay was employed using recombinant Influenza A H1N1 (A/California/04/2009) neuraminidase and the substrate 2′-(4-methylumbelliferyl)-α-D-N-acetylneuraminic acid (MUNANA). Before testing the compounds, the assay conditions were optimized by performing titration experiments with increasing enzyme units (0.01–1.0 U). Fluorescence signals increased proportionally with the enzyme amount confirming the linear range of the reaction. Based on these data, 0.1 U of recombinant NA was selected as the working concentration, representing the optimal balance between signal intensity and reaction linearity (Figure 7a). The reliability and sensitivity of the fluorometric assay were validated using oseltamivir (OSELT) as the reference inhibitor, which yielded an IC_50_ value of 0.36 µM, thereby confirming the robustness of the optimized MUNANA-based assay (Figure 7b).

**Figure 8:**
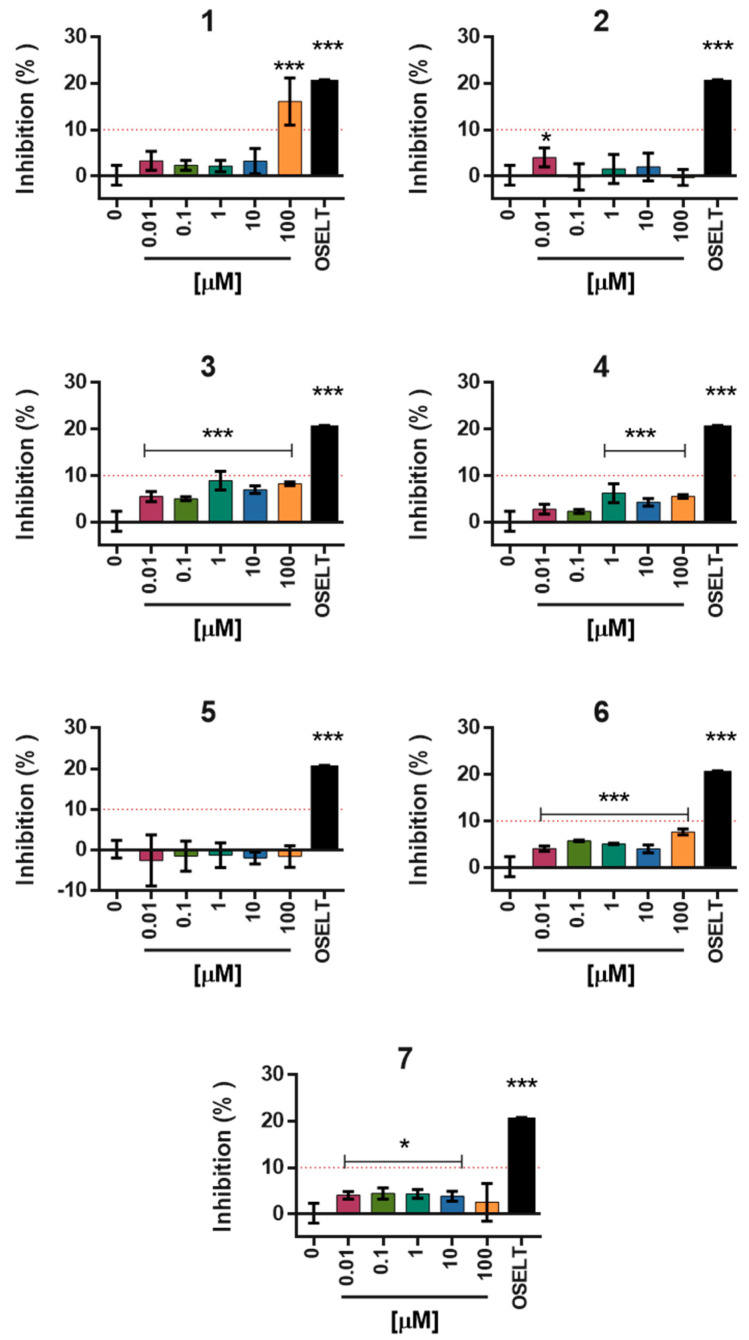
Fluorometric evaluation of neuraminidase inhibition by the selected compounds **1**–**7** tested at concentrations ranging from 0.01 to 100 µM. The numbers **1**–**7** identified the respective tested compounds. Oseltamivir at 0.1 µM was included as an internal reference inhibitor (black bars). Fluorescence data were processed to determine the inhibition percentage relative to the uninhibited enzyme control (0% inhibition). Results are expressed as mean ± SD of three independent experiments performed in duplicate. Statistical significance was assessed by one-way ANOVA with Bonferroni’s post hoc test (* *p* < 0.05; *** *p* < 0.001 vs. 0 control).

Under these validated conditions, all seven candidate compounds **1**–**7** were tested over a concentration range of 0.01–100 µM. As part of the standard assay workflow, fluorescence background controls (substrate + compound without enzyme, and compound alone) were routinely included for each concentration tested. The aggregated data, presented in Supplementary Materials in Figure S14, confirmed the absence of compound autofluorescence or substrate quenching, indicating that the inhibitory signals reported are not influenced by fluorescence interference. Oseltamivir was included as an internal reference control at 0.1 µM to ensure assay consistency across independent experiments. At this concentration, oseltamivir produced approximately 20% inhibition of NA activity, a value consistent with its expected dose–response profile. Although some compounds yielded statistically significant differences compared to the control, only inhibitory effects exceeding 10% were considered biologically relevant. Among the tested molecules, compound **1** consistently exhibited a reproducible inhibitory trend, showing mean inhibition values above 15% at 100 µM, whereas all other compounds displayed weak or negligible inhibition, with values below 10% (Figure 8).

**Figure 9:**
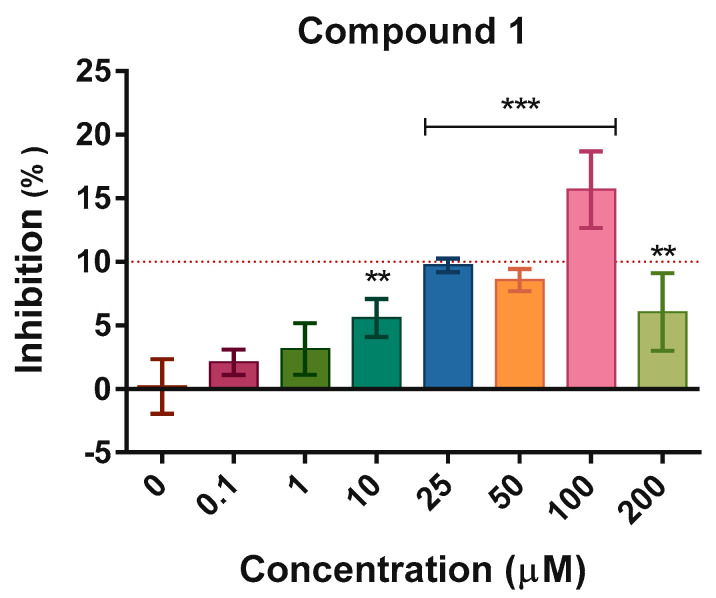
Screening of compound **1** against neuraminidase (NA) enzymatic activity using the fluorescent MUNANA assay. Serial twofold dilutions of compound **1** were tested from 200 to 25 µM, followed by tenfold dilutions from 10 to 0.1 µM. Data are expressed as percentage of inhibitory activity relative to the uninhibited enzyme control (0 concentration) and represent mean ± SD from three independent experiments performed in duplicate. Asterisks denote significant differences, with ** *p* < 0.01 and *** *p* < 0.001 compared to the uninhibited control.

Given its reproducible activity profile, compound **1** was further evaluated over an extended concentration range (0.01–200 µM), including additional intermediate doses to better define its dose–response relationship. Compound **1** exhibited a clear concentration-dependent inhibition curve, with inhibition values gradually increasing from baseline at 0.01 µM to a maximum mean inhibition of approximately 14–15% confirmed at 100 µM. At higher concentrations, however, a decrease in inhibitory activity was observed, suggesting a possible partial loss of specificity or reduced assay stability at the upper range (Figure 9). Nonlinear regression yielded an apparent IC_50_ far beyond the highest concentration tested (IC_50_ > 1 mM), indicating that inhibition by compound **1** was extremely weak and that the fitted value was entirely extrapolated. For this reason, we primarily described its effect in terms of percentage inhibition at each tested concentration.

Taken together, these results confirmed the feasibility of the assay and indicated that, within the tested chemical series, compound **1** exerted a modest but reproducible inhibitory activity against recombinant H1N1 neuraminidase at non-cytotoxic concentrations. The trend supported the hypothesis of a specific though low-affinity interaction with the NA catalytic pocket, consistent with computational predictions and providing a rationale for further structure–activity optimization.

## 3. Materials and Methods

### 3.1. Molecular Modeling

#### 3.1.1. Protein Structure Preparation and Mutagenesis

The crystal structures of neuraminidase (NA) from influenza A (H1N1) virus, strain A/California/04/2009, co-crystallized with the antiviral drugs laninamivir, zanamivir, and oseltamivir (PDB IDs: 3TI3, 3TI5, and 3TI6, respectively), 10.1371/journal.ppat.1002249 along with the NA structure from strain A/Michigan/45/2015 (PDB ID: 7S0I) [31] were retrieved from the RCSB Protein Data Bank (https://www.rcsb.org/, accessed on 18 March 2025). To align structural modeling with biological assays, we replaced the following amino acid into PDB structures 3TI3, 3TI5, and 3TI6 to reflect the sequence of the A/Michigan/45/2015 strain (PDB 7S0I): N199S, V240I, N247D, V263I, N269K, I314M, I321V, N372K, N389K, and K432E. Therefore, protein structures were processed using the Protein Preparation Wizard [53] of the Schrödinger Suite, applying the OPLS_2005 force field, including assigning bond orders, generating missing side chains, optimizing hydrogen bonding networks, and adjusting protonation states at a simulation pH of 7.4 as predicted by PROPKA. Non-essential water molecules and heteroatoms diverse from the co-crystallized antiviral ligands were removed. Subsequently, the in silico mutated protein structures were subjected to energy minimization using the Minimization tool of MacroModel [39]. A two-step minimization protocol was applied as follows. In the first step, only the ten mutated residues were energy minimized, with the rest of the protein restrained using a harmonic force constant of 200 kJ/mol. In the second step, minimization was extended to include all atoms within 2 Å of the mutated residues. Minimization was performed using the PRCG (Polak–Ribiere Conjugate Gradient) algorithm with OPLS_2005 as the force field and water as the implicit solvent. A limit of 10,000 iterations was established, with convergence based on gradient minimization using a threshold of 0.05.

#### 3.1.2. Molecular Dynamics Simulations

To refine the mutated protein–ligand complexes, an ultimate minimization was performed via short molecular dynamics (MDs) simulations using the Desmond module [40] within the Schrödinger Suite. Specifically, residues undergoing mutation and those within a 2 Å radius were subjected to energy minimization and a 1.2 ns MDs run. The remaining regions of the protein were positionally restrained using a harmonic force constant of 50 kcal/mol/Å^2^ to preserve the overall structure. All simulations were conducted in NPT ensemble at 300 K and 1 atm. Temperature and pressure control were regulated using a Nose–Hoover thermostat and a Martyna–Tobias–Klein barostat, with relaxation times of 1.0 ps and 2.0 ps, respectively. A time step of 2.0 fs was applied throughout the simulations. Each protein–ligand complex was embedded in a 1000 Å^3^ orthorhombic box and solvated using the explicit TIP3P water model. System neutralization and physiological ionic strength (0.15 M) were achieved through the addition of appropriate numbers of sodium and chloride counterions. System interactions were described using the OPLS_2005 force field, applying a 9 Å cutoff for short-range Coulombic interactions. Prior to production runs, the “Relax model system before simulation” option was enabled, ensuring proper equilibration through initial minimization and short MD simulations under NPT conditions. Following system relaxation and preparation, full MD simulations were performed for 100 ns for each complex. Trajectories were recorded at 100 ps intervals, yielding 1000 frames per system. Post-simulation analyses focused on evaluating protein–ligand complex stability, as well as the frequency and nature of molecular interactions throughout the trajectory.

#### 3.1.3. Pharmacophore Modeling Design and Virtual Screening

The mutated proteins in complex with laninamivir, zanamivir, and oseltamivir were used to create three structure-based pharmacophore models by LigandScout (v 4.5) [41]. Pharmacophores were then aligned and combined in one hypothesis using the function “generate shared feature pharmacophore”. Virtual screening was performed according to the follow parameters: Pharmacophore-Fit as scoring function, “get best matching conformation”, and “check exclusion volumes” option set as “on” as retrieval mode. Additionally, the in-house library was screened with “max. number of omitted features” set to 1. All databases were prepared using the IconBest option, leading to 200 conformers for each compound.

#### 3.1.4. Library Validation

To validate the pharmacophore model, a library including active and inactive compounds was developed. Data were retrieved from BindingDB (https://www.bindingdb.org/, accessed on 26 March 2025), including only compounds tested on H1N1 strain with reported IC_50_ value, for a total of 258 compounds. The dataset was cleaned by using a KNIME workflow (v 5.4.3). We retained only IC_50_ entries with exact equality relationships (“=”) and converted all structures to canonical SMILES using RDKit for preprocessing. Duplicate entries were identified based on identical canonical SMILES representations. Mean and standard deviation were calculated for the duplicated, i.e., molecules with more than one activity value reported on the same target. Only compounds with standard deviations below 20% of their average IC_50_ values were retained for analysis. Therefore, mean was recalculated to obtain the final mean IC_50_ value. Molecules were defined as active with IC_50_ values < 10 nM and inactive with IC_50_ values > 10,000 nM. Compounds with activity records between 10 and 10,000 nM were discarded from the validation set. Such a procedure resulted in 87 active and 508 inactive compounds.

#### 3.1.5. Ligand Preparation

All compounds were processed through LigPrep (Schrödinger Suite, release 2021-4: LigPrep, Schrödinger, LLC, New York, NY, USA, 2021) at pH 7.4 to replicate physiological conditions. Molecular geometries were refined using OPLS_2005 parameters, with chirality preserved according to input configurations.

#### 3.1.6. ADMET Properties Prediction

ADMET analysis of the virtual screening hits was performed using the Percepta tool [42] of ACD/Labs software (Version 2025). Specifically, lead-like properties, compliance with Lipinski’s rules, and aqueous solubility were calculated. Compounds were retained if they were classified as “good” for both lead-like and Lipinski criteria and as soluble for aqueous solubility.

#### 3.1.7. Flexible Docking Studies

Flexible docking simulations were performed by GOLD (v 2024.0.1) [43] using the crystal structure of N1 bound to zanamivir (PDB ID: 3TI5) to identify the most favorable binding interaction. The side chains of residues S246, D247, E276, and N347 were treated as flexible and allowed to rotate according to the internal rotamer library available in the GOLD Suite. The docking grid was defined to encompass all residues within a 12 Å radius from a central point determined by the average coordinates of three co-crystallized ligands (x = 29.8, y = 14.79, z = –20.96). GoldScore was employed as the primary scoring function, while ASP was used for rescoring. All docking simulations were carried out using the standard default parameters, with each ligand subjected to 10 genetic algorithm runs. The “allow early termination” option was disabled. Results differing by less than 0.8 Å in ligand-all atom RMSD were clustered together. To evaluate the reliability of the flexible docking approach, the Root Mean Square Deviation (RMSD) was used as a validation metric. For further analysis and visualization, the top-ranked pose for each ligand based on the ASP score was selected. All docking results were visualized using BIOVIA Discovery Studio (BIOVIA, Dassault Systèmes, [Discovery Studio Client] [2025], Dassault Systèmes [2025], San Diego, CA, USA).

#### 3.1.8. Chemical Characterization of Compounds **1**–**7**

Our repository CHIME collected 1683 compounds (CHIME2024), which have been obtained from fine chemical suppliers or already synthesized by us. In detail, indole-based compounds **1**–**4** were obtained from Merck Sigma Aldrich (Milano, Italy); the synthetic procedures to obtain isoquinoline-based compounds **5**–**7** were reported in our previous papers as detailed in Experiments Sections. The seven studied compounds possessed >95% of purity and were characterized by NMR recorded on Varian Gemini 500 (Siemens Healthineers, Palo Alto, CA, USA) in DMSO *d*_6_. Melting points were recorded on Buchi B-545 (BUCHI Labortechnik AG, Flawil, Switzerland). The chemical stability was assessed in phosphate buffer solution (PBS, pH = 7.4) from stock solutions prepared in DMSO (20 mM concentration). The stock solutions were stored protected from light at room temperature for four days. Subsequently, the stock solutions were diluted 1-fold with PBS to obtain working solutions that were incubated for 4 h at 37 °C. We preliminarily evaluated the stability by using reverse-phase thin layer chromatography (RP-TLC) on pre-coated TLC-plates of RP18-modified silica gel 60 F254 (5 cm × 10 cm, E. Merck, Darmstadt, Germany). Linear ascending development was carried out in a chromatographic tank in acetone/water (2:1 *v*/*v*) eluant. The developed plates were analyzed at 254 nm for UV detection through visual inspection that revealed that the studied compounds preliminarily demonstrated chemical stability in PBS.

1-(1*H*-Indol-3-ylcarbonyl)-3-piperidinecarboxylic acid (**1**, CAS Registry Number: 1016752-59-2): Off-white powder soluble in DMSO. M.p. 205 dec ^1^H NMR (500 MHz, DMSO-*d*_6_) δ ppm 1.43–1.46 (m, 1 H, CH), 1.62–1.66 (m, 2 H, CH), 1.69–1.97 (m, 1 H, CH), 1.98–2.00 (m, 1 H, CH), 3.00–3.12 (m, 2 H, CH), 4.03–4.06 (m, 1 H, CH), 4.27–4.30 (m, 1 H, CH), 7.07–7.15 (m, 2 H, ArH), 7.42 (d, *J* = 7.8 Hz, 1 H, ArH), 7.63 (d, *J* = 8.3 Hz, 1 H, ArH), 7.65 (d, *J* = 2.5 Hz, 1 H), 11.54 (br s, 1 H, NH), 12.36 (br s, 1 H, COOH).

3-[(1*H*-Indol-3-yl)formamido]propanoic acid (**2**, CAS Registry Number: 171817-97-3): Off-white powder soluble in DMSO. M.p. 157–159 °C ^1^H NMR (500 MHz, DMSO-*d*_6_) δ ppm 2.50–2.61 (m, 2H, CH_2_) 3.43–3.45 (m, 2 H, CH_2_) 7.05–7.13 (m, 2 H, ArH), 7.38–7.40 (m, 1 H, ArH), 7.91 (br s, 1 H, ArH), 7.96 (m, 1 H, ArH), 8.09 (br s, 1 H, CONH), 11.49 (br s, 1 H, NH), 12.20 (br s, 1 H, COOH). ^13^C NMR (126 MHz, DMSO-*d*_6_) δ ppm 34.8, 35.3, 111.0, 112.2, 120.8, 121.4, 122.3, 126.5, 128.2, 136.5, 165.1 (CO) 173.6 (COOH).

γ-Oxo-1H-indole-3-butanoic acid (**3**, CAS Registry Number: 835-45-0): White powder soluble in DMSO. M.p. 235 °C dec. ^1^H NMR (500 MHz, DMSO-*d*_6_) δ ppm 2.63–2.66 (m, 2 H, CH_2_), 3.24–3.26 (m, 2 H, CH_2_), 6.74 (d, *J* = 3.4 Hz, 1 H, ArH), 7.23–7.32 (m, 2 H, ArH), 7.61 (d, *J* = 7.3 Hz, 1 H), 7.93 (d, *J* = 3.9 Hz, 1 H, NH), 8.31 (d, *J* = 8.31 Hz, 1 H), 12.27 (br s, 1 H, COOH).

4-Methoxy-1*H*-indole-3-carboxylic acid (**4**, CAS Registry Number: 203937-50-2): Yellow powder soluble in DMSO. M.p. 162 °C dec. ^1^H NMR (500 MHz, DMSO-*d*_6_) δ ppm 3.91 (s, 3 H, OCH_3_), 6.71 (d, *J* = 7.3 Hz, 1 H, ArH), 7.09–7.14 (m, 2 H, ArH), 7.95 (d, *J* = 2.4 Hz, 1 H, CH), 11.55 (br s, 1 H, COOH), 11.91 (br s, 1 H, NH).

(3*R*,4*S*)-2-[(4-Chlorophenyl)methyl]-1,2,3,4-tetrahydro-3-(4-methoxyphenyl)-1-oxo-4-isoquinolinecarboxylic acid [54] (**5**, CAS Registry Number: 745814-32-8): M.p. 258–160 °C. White powder soluble in DMSO. ^1^H NMR (500 MHz, DMSO-*d*_6_) δ ppm 3.65 (s, 3H, OCH_3_), 3.84 (d, *J* = 15.1 Hz, 1 H, CH), 4.03 (s, 1 H, CH), 5.18–5.22 (m, 2 H, 2 CH), 6.77–6.79 (m, 2 H, ArH), 6.92–6.94 (m, 2 H, ArH), 7.19–7.20 (m, 1 H, ArH), 7.29–7.30 (m, 3 H, ArH), 7.42 (dd, *J* = 7.4 and 1.8 Hz, 2 H, ArH), 7.96 (dd, *J* = 7.4 and 1.8 Hz, 1H, ArH). ^13^C NMR (126 MHz, DMSO-*d*_6_) δ ppm 49.0, 51.3, 55.5, 61.1, 114.5, 127.4, 127.7, 128.3, 128.5, 129.3, 130.0, 130.4, 131.2, 132.1, 132.5, 134.2, 136.9, 159.1, 163.8 (CO), 172.5 (COOH).

3,4-Dihydro-6,7-dimethoxy-1-(1-methylethyl)-2(1*H*)-isoquinolinesulfonamide [55] (**6**, CAS Registry Number: 1215113-23-7): White powder soluble in DMSO. M.p. 187–190 °C. ^1^H NMR (500 MHz, DMSO-*d*_6_) δ ppm 0.86−0.89 (m, 6H, CH_3_), 1.97−2.01 (m, 1H, CH), 2.58−2.61 (m, 1H, CH), 2.87−2.89 (m, 1H, CH), 3.33−3.39 (m, 1H, CH), 3.51−3.53 (m, 1H, CH), 3.70 (s, 6H, OCH_3_), 4.27 (d, *J* = 7.1 Hz, 1H, CH), 6.56 (br s, 2H, NH_2_), 6.68 (s, 1H, ArH), 6.69 (s, 1H, ArH).

1-Cyclopentyl-3,4-dihydro-6,7-dimethoxy-2(1*H*)-isoquinolinesulfonamide [55] (**7**, CAS Registry Number: 1215113-28-2): White powder soluble in DMSO. M.p. 193–195 °C. ^1^H NMR (500 MHz, DMSO-*d*_6_) δ ppm 1.42−1.69 (m, 8H, CH), 2.05−2.07 (m, 1H, CH), 2.56–2.60 (m, 1H, CH), 2.91−2.94 (m, 1H, CH), 3.33−3.38 (m, 1H, CH), 3.57−3.59 (m, 1H, CH), 3.69 (s, 6H, OCH_3_), 4.31 (brs, 1H, CH), 6.53 (brs, 2H, NH_2_), 6.67 (s, 1H, ArH), 6.68 (s, 1H, ArH).

### 3.2. Biological Assay

Human larynx epithelial HEp-2 cells (ATCC CCL-23, Manassas, VA, USA) were cultured in RPMI-1640 medium (Euroclone, Milan, Italy) supplemented with 10% fetal bovine serum (FBS), 2 mM L-glutamine, 100 U/mL penicillin, and 100 µg/mL streptomycin, and maintained at 37 °C in a humidified 5% CO_2_ atmosphere.

Cytotoxicity was evaluated by the MTS colorimetric assay (CellTiter 96 Aqueous One Solution, Promega, Madison, WI, USA) as previously described [56]. Briefly, HEp-2 cells were seeded in 96-well plates, exposed to serial dilutions of compounds for 24 h, and incubated with MTS reagent. Absorbance was measured at 490 nm using an iMark Microplate Absorbance Reader (Bio-Rad, Hercules, CA, USA). CC_50_ values were calculated as the concentration reducing metabolic activity by 50% relative to untreated controls.

Oseltamivir phosphate (Thermo Scientific Chemicals, Waltham, MA, USA, #461170010), used as reference neuraminidase inhibitor, was dissolved in DMSO (25 mM) and briefly sonicated (5 s). Test compounds (**1**–**7**) were solubilized in DMSO at 12.5–50 mM according to their solubility; compounds **5** and **6** required short ultrasonic cycles. Stock solutions were diluted to the desired concentrations, keeping DMSO ≤ 0.5% (*v*/*v*) in all assays.

Neuraminidase (NA) activity was evaluated with the NA-Fluor™ Influenza Neuraminidase Assay Kit (Invitrogen™, Thermo Fisher Scientific, Cat, Waltham, MA, USA, #4457091) using recombinant H1N1 NA (A/California/04/2009) expressed in HEK293 cells (Sino Biological, Wayne, PA, USA, #11058-VNAHC). Reactions were performed in 96-well black plates using the MUNANA substrate, and fluorescence was recorded at 365/445 nm (excitation/emission). Oseltamivir (IC_50_ = 0.36 ± 0.03 μM, n = 3) served as positive control. Results were expressed as percentage inhibition of enzymatic activity versus the uninhibited control. To exclude fluorescence interference by tested compounds, background fluorescence was evaluated for each molecule at the highest concentrations used in the enzymatic assay (from 1 µM up to the maximal tested concentration). Control wells contained (i) compound + MUNANA substrate without neuraminidase and (ii) compound alone without substrate. Background values were subtracted from the raw fluorescence of the corresponding inhibition assays, according to the manufacturer’s recommendations. No autofluorescence or substrate fluorescence quenching was observed for any compound.

All experiments were carried out in at least three independent replicates. Data analysis and CC_50_/IC_50_ calculations were performed with GraphPad Prism v. 8.0 (GraphPad Software, San Diego, CA, USA) using one-way ANOVA followed by Bonferroni’s post hoc test. For oseltamivir, IC_50_ values were obtained by nonlinear regression using a four-parameter logistic model (GraphPad Prism, v. 8.0). For compound **1**, which displayed a shallow inhibition curve that did not reach 50% inhibition within the concentration range explored, the IC_50_ was treated as an extrapolated apparent estimate and reported qualitatively (IC_50_ > 1 mM), rather than as a precise quantitative parameter.

## 4. Conclusions

We applied a computational strategy as a valuable tool for a virtual screening campaign in our database of small molecules already available for testing purposes, thus reducing synthetic efforts and applying fast and cost-effective protocols. The combination of a 2015-informed computational model with a 2009-derived experimental enzyme offered a coherent and complementary approach to validate the predicted inhibitory trends within the conserved N1 active site of NA. This applied multi-step in silico study included the development of a structure-based pharmacophore model, subsequently validated through docking and molecular dynamics simulations. The in silico-driven approach guided the prioritization of seven compounds with favorable chemical features as well as desired drug-like properties. All tested compounds showed a favorable cytotoxicity profile in epithelial cells, with CC_50_ values higher than 100 µM, confirming their suitability for enzymatic testing. The fluorometric assay, validated using oseltamivir (IC_50_ = 0.36 µM), identified only one indole-based compound (i.e., compound **1**) displaying a modest yet reproducible inhibitory activity (~15% inhibition at 100 µM) without cytotoxic effects, thus representing a prototype for optimization toward active neuraminidase inhibitors. Although its potency was limited, the compound reproduced key interactions predicted in silico, supporting the reliability of the computational model and suggesting its potential use as a chemical template for further optimization. Some limitations should be acknowledged. The modest inhibitory effect observed indicated that additional structural refinement of the identified hit compound **1** was needed to enhance affinity and potency. Nevertheless, the use of recombinant neuraminidase from the 2009 A/California/04 strain, although not encompassing the full conformational variability of more recent N1 variants, was coherent with the hybrid computational model adopted in this study. By introducing the amino acid substitutions of the 2015 A/Michigan/45 strain into the 2009 crystallographic framework, the model captured the adaptive features of contemporary N1 enzymes while preserving the experimentally validated backbone required for accurate molecular simulations. The use of the 2009-derived recombinant enzyme for biological validation therefore ensured a consistent experimental counterpart to the computational predictions. Finally, while the enzymatic inhibition assay provided an indirect measure of NA interference and cannot discriminate among distinct inhibitory mechanisms, the observed reproducible inhibition supported the predictive robustness of the integrated workflow. Overall, this work established a coherent and validated platform that combines rational computational design with targeted biological evaluation. This study provides a clear proof of concept for the predictive capacity of the adopted structure-based strategy and identifies an indole–amide scaffold as a starting point for further structure optimization toward next-generation neuraminidase inhibitors. 

## Figures and Tables

**Table 1:** Compounds **1**–**7** identified from the virtual screening procedure along with their matching features as well as their superposition to the pharmacophore model: hydrogen bond acceptors (HA_1_-HA_2_, red arrows/red square), hydrogen bond donor (HD1, green sphere/green square) negatively ionizable (NI_1_) red stars).

Compounds	2D Structure	Pharmacophore Mapping	Matched Features
**1**	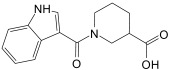	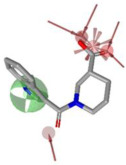	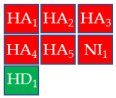 Fit score: 77.38
**2**	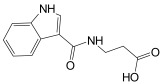	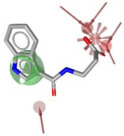	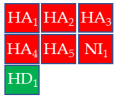 Fit score: 75.91
**3**	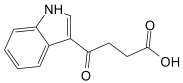	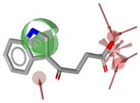	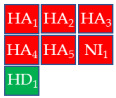 Fit score: 75.56
**4**	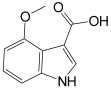	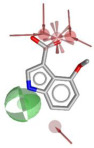	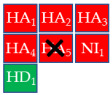 Fit score: 66.2
**5**	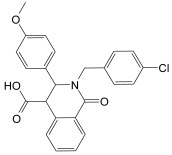	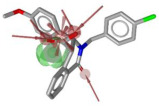	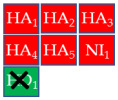 Fit score: 67.96
**6**	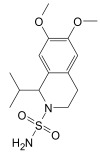	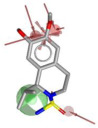	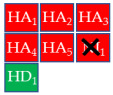 Fit score: 66.33
**7**	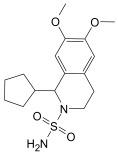	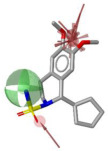	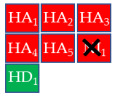 Fit score: 65.50

**Table 2:** Cytotoxicity (CC_50_ values) of NA-targeting compounds determined by MTS assay in HEp-2 cells.

Compound	CC_50_ (µM)
**1**	**^1^ **N.D.
**2**	**^1^ **N.D.
**3**	670.6
**4**	244.2
**5**	152
**6**	534
**7**	**^1^ **N.D.

^1^ N.D. (not detected): for these compounds, cell viability values remained comparable to those of the vehicle control up to 100 µM, thus not allowing the calculation of a CC_50_ value.

## Data Availability

Data will be made available from the authors upon request.

## Supplementary Materials

The following supporting information can be downloaded at https://www.mdpi.com/article/10.3390/molecules30234636/s1, Figure S1: RMSD plots for the mutated X-ray protein–ligand complexes of 3TI3 (A), 3TI5 (B), and 3TI6 (C). Figure S2: Interaction profiles of N1 bound to laninamivir (A,D), zanamivir (B,E), and oseltamivir (C,F) observed during the simulations. (A–C). Figure S3: Conformational comparison of N1 complexes: alignment of PDB entries, along with a focus on the binding site (A). Histogram showing RMSD (in Å) calculated on protein atoms (B). Figure S4. Superimposition of co-crystallized ligands and corresponding flexible residues (both shown as sticks in the same color) with predicted docking poses. Figures S5–S13: NMR spectra for compounds **1–7**. Table S1: Estimated physicochemical properties and drug-likeness by Percepta platform (ACD/Labs). Figure S14: Fluorescence interference controls for NA assay of compounds **1–7**.

## Abbreviations

The following abbreviations are used in this manuscript:

ACD	Advanced chemistry development
ADMET	Absorption, distribution, metabolism, excretion, and toxicity
ANOVA	Analysis of variance
AUC	Area under curve
DANA	2-deoxy-2,3-dehydro-N-acetylneuraminic acid
DMSO	Dimethyl sulfoxide
EF	Enrichment factor
EMA	European Medicine Agency
FDA	Food and Drug Administration
HA	Hemagglutinin
IAV	Influenza A virus
MD	Molecular dynamic
MTS	3-(4,5-dimethylthiazol-2-yl)-5-(3-carboxymethoxyphenyl)-2-(4-sulfophenyl)-2H-tetrazolium
MUNANA	4-Methylumbelliferyl-N-acetyl-α-D-neuraminic acid
NA	Neuraminidase
NAIs	Neuraminidase inhibitors
PA	Polymerase acid protein
PB1	Polymerase basic protein 1
PB2	Polymerase basic protein
PDB	Protein Data Bank
RMSD	Root mean square deviation
RP-TLC	Reverse-phase thin layer chromatography
ROC	Receiver operating characteristic
VS	Virtual screening
