# Supplementary material for: Discovery of Influenza Neuraminidase Inhibitors: Structure-Based Virtual Screening and Biological Evaluation of Novel Chemotypes
Source: Molecules. 2025 Dec 2;30(23):4636. doi: 10.3390/molecules30234636 (PMC12693396; doi:10.3390/molecules30234636)
Supplement: Supplementary file 1 [file molecules-30-04636-s001.zip › molecules-3989593-supplementary.pdf]

Supplementary Material

Discovery of Influenza Neuraminidase Inhibitors: Struc-ture-Based Virtual Screening and Biological Evaluation of Novel Chemotypes

Rosaria Gitto,[1,*] Lisa Lombardo, [1] Angela Ravenda, [1] Francesco Broccolo, [2] Antonio Mastino, [3] Laura De Luca,[1] Francesca Marino-Merlo[1]

[1] CHIBIOFARAM Department, University of Messina, Viale F. d'Alcontres 31, I-98166 Messina, Italy
[2]DiMeS Department of Experimental Medicine, University of Salento, Lecce, Italy
[3]The Institute of Translational Pharmacology, CNR, Via Fosso del Cavaliere 100, Roma 00133, Italy.

*Correspondence: rgitto@unime.it

Contents:

1. **Molecular dynamics simulations (Figures S1-S2)**
2. **Flexible docking studies (Figures S3-S4)**
3. **Estimated physicochemical properties (Table S1)**
4. **NMR spectra for studied compounds 1-7 (Figures S5-S13)**
5. **Fluorescence interference controls for NA assay (Figure S14)**

1. **Molecular dynamics simulations**

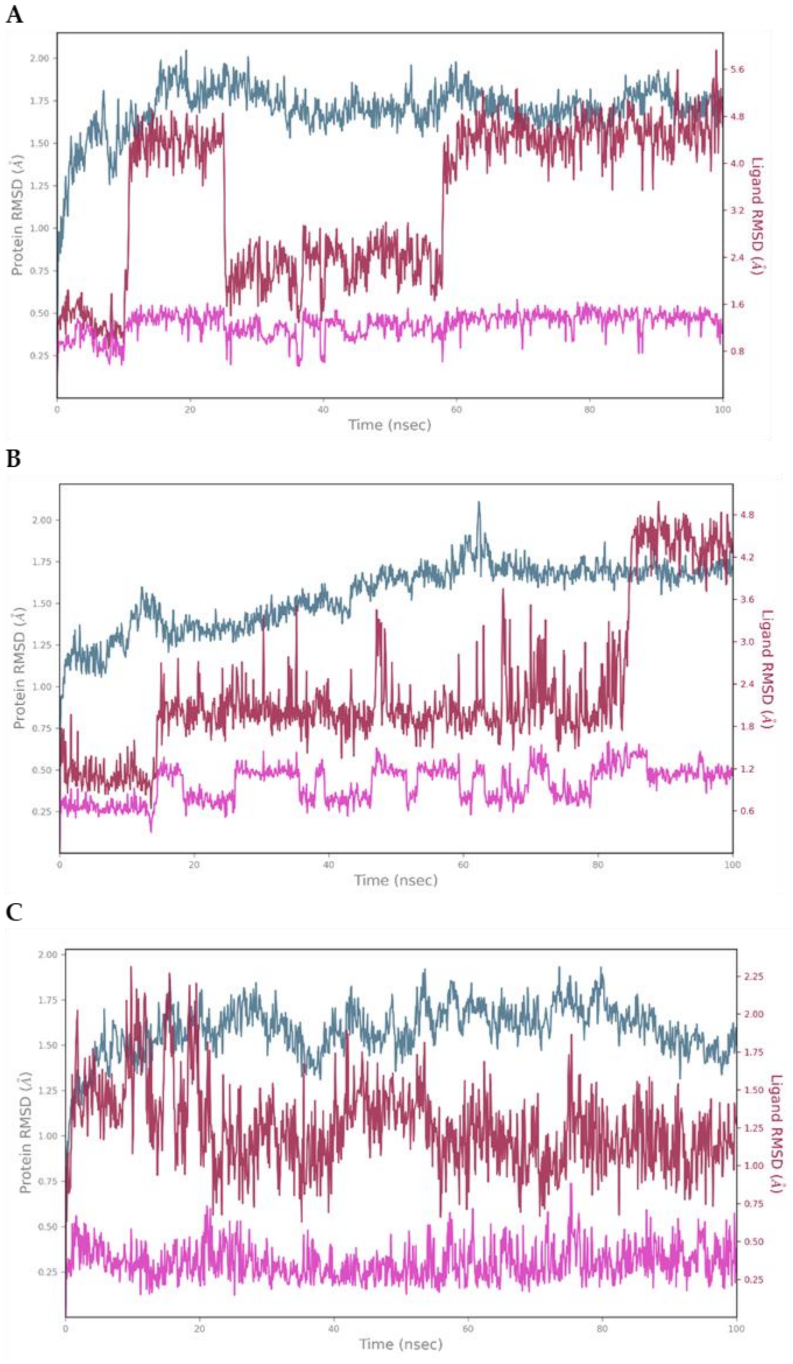

Figure S1. RMSD plots for the mutated X-ray protein–ligand complexes of 3TI3 (A), 3TI5 (B), and 3TI6 (C). The x-axis represents simulation time (frames). The left y-axis corresponds to protein backbone RMSD values (Å), while the right y-axis corresponds to ligand RMSD values (Å). Blue plots indicate protein backbone fluctuations, burgundy plots depict ligand stability within the binding site, and fuchsia plots represent ligand RMSD values, all calculated with respect to the frame at time 0.

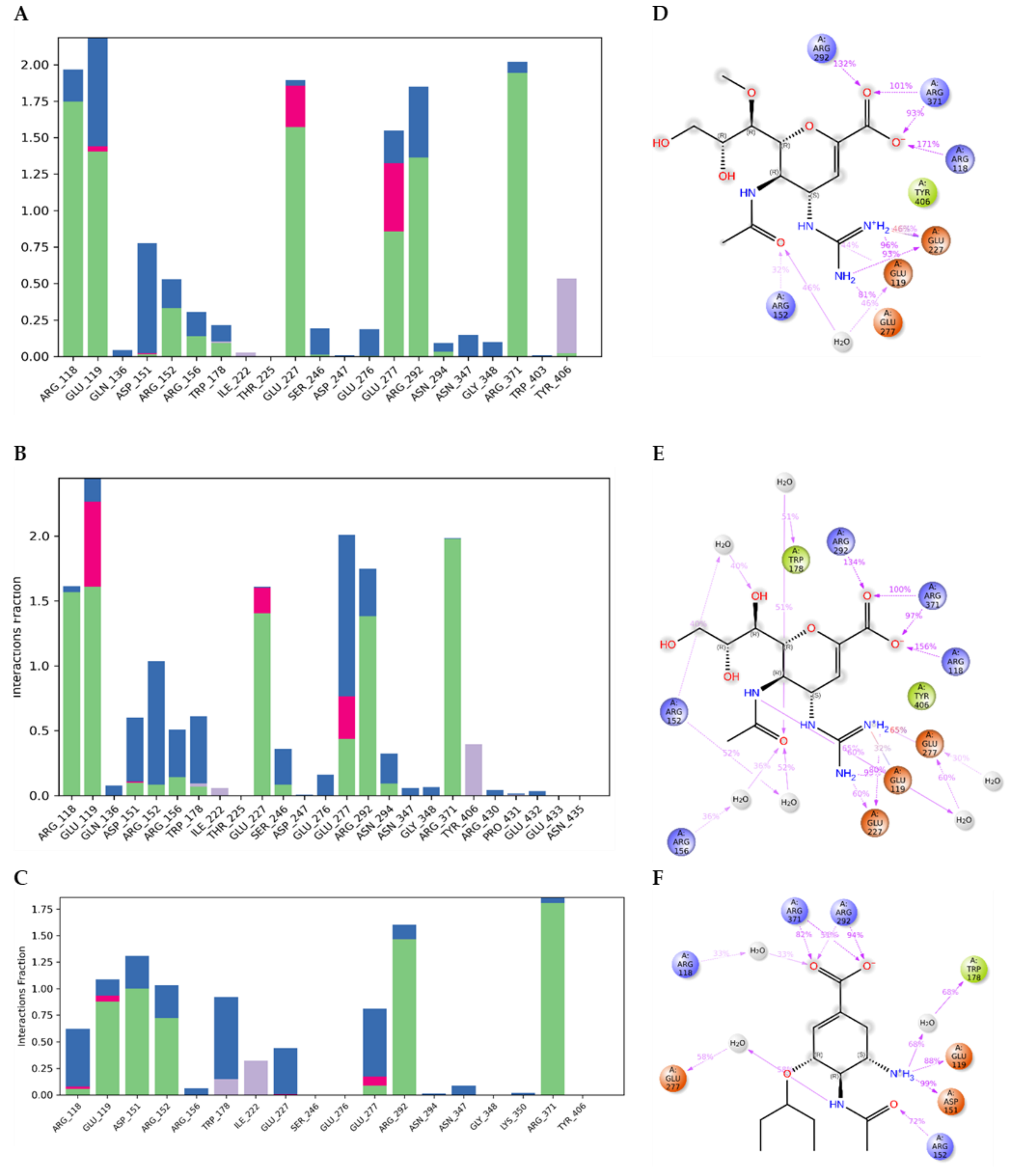

Figure S2. Interaction profiles of N1 bound to laninamivir (A, D), zanamivir (B, E), and oseltamivir (C, F) observed during the simulations. (A-C) Bar plots summarizing protein-ligand interactions, classified as hydrogen bonds (green), hydrophobic contacts (purple), ionic interactions (fuchsia), and water bridges (blue). (D-F) Detailed maps of ligand-residue contacts, showing only interactions occurring in more than 30% of the simulation time.

2. Flexible docking studies

Comparison of the three PDB complexes revealed a highly conserved conformation of residues in the catalytic site (Figure S3A), except for S246, D247 (N247 in the wild-type protein), E276, and N347. This variation is consistent with the presence of distinct functional groups in the pharmacophore model, which appear to induce a positional shift of E276 (Figure S3). RMSD calculations on protein atoms yielded values below 1.3 Å. Considering both the high resolution of the available PDB crystal structures (overall RMSD 1.7-1.9 Å) and the lower RMSD values obtained when comparing 3TI5 with 3TI3 and 3TI6, 3TI5 was selected as the protein model for subsequent docking studies (Figure S3).

A

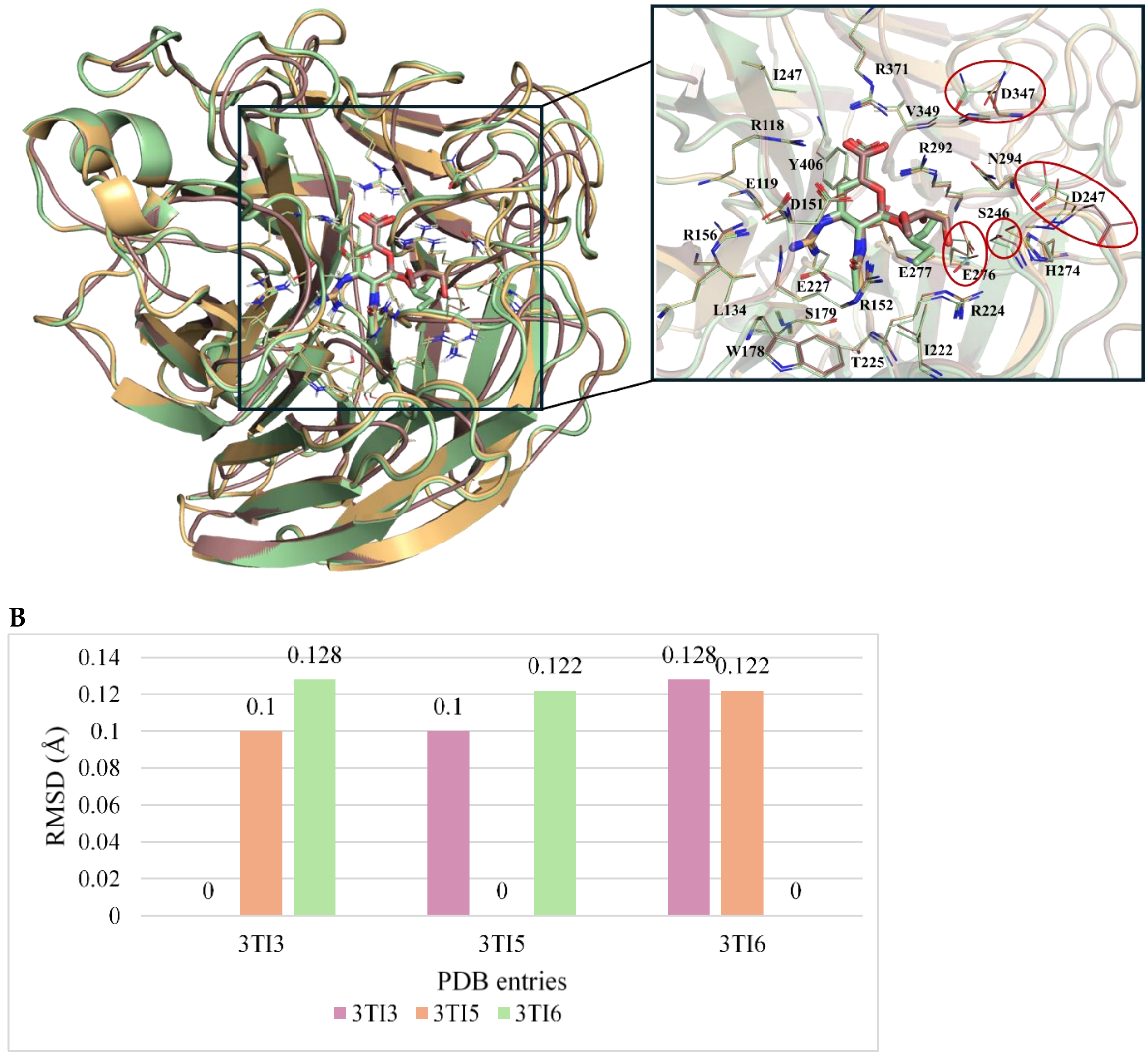

Figure S3. Conformational comparison of N1 complexes: alignment of PDB entries, along with a focus on the binding site (A). Histogram showing RMSD (in Å) calculated on protein atoms (B).

The docking protocol was validated by redocking the co-crystallized ligands of the selected X-ray complexes, allowing flexibility in the corresponding binding site residues. The RMSD values between the

predicted and crystallographic poses were 0.917 Å for Laninamivir (Figure S4A), 1.07 Å for Zanamivir (Figure S4B), and 0.611 Å for Oseltamivir (Figure S4C), confirming the accuracy and predictive power of the protocol. Moreover, rescoring improved pose predictiveness, as reflected by the same trend observed when comparing the original ligand conformations with the results obtained for flexible residues (Figure S4).

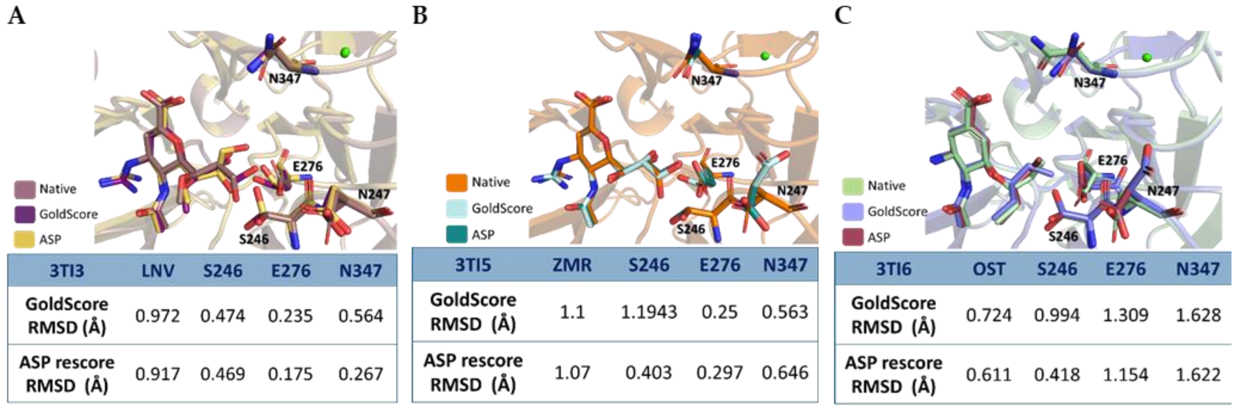

3TI3	LNV	S246	E276	N347
GoldScore RMSD (Å)	0.972	0.474	0.235	0.564
ASP rescore RMSD (Å)	0.917	0.469	0.175	0.267

3TI5	ZMR	S246	E276	N347
GoldScore RMSD (Å)	1.1	1.1943	0.25	0.563
ASP rescore RMSD (Å)	1.07	0.403	0.297	0.646

3TI6	OST	S246	E276	N347
GoldScore RMSD (Å)	0.724	0.994	1.309	1.628
ASP rescore RMSD (Å)	0.611	0.418	1.154	1.622

Figure S4. Superimposition of co-crystallized ligands and corresponding flexible residues (both shown as sticks in the same color) with predicted docking poses. (A) Laninamivir and its flexible residues (dirty violet) compared with the best GoldScore pose (purple) and ASP pose (yellow). (B) Zanamivir and its flexible residues (orange) compared with the best GoldScore pose (cyan) and ASP pose (deep teal). (C) Oseltamivir and its flexible residues (pale green) compared with the best GoldScore pose (light blue) and ASP pose (raspberry).

3.Estimated selected physicochemical properties and drug-likeness

Table S1. Physicochemical and drug-likeness parameters calculated by Percepta suite 14.55.0 (ACDLabs, https://www.acdlabs.com/) for compounds **1-7**

Structure	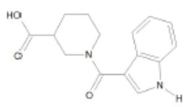	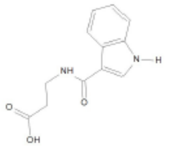	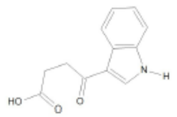	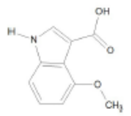	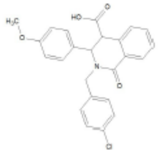	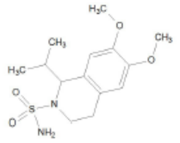	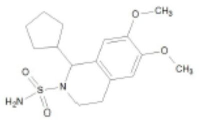
*ID	1	2	3	4	5	6	7
Caco-2	Moderately pe...	Poorly perme...	Moderately pe...	Highly permea...	Highly permea...	Highly permea...	Highly permea...
PPB	Extensively b...	Moderately bo...	Extensively b...	Strongly bound	Extensively b...	Moderately bo...	Moderately bo...
CNS	Non-penetrant	Non-penetrant	Non-penetrant	Non-penetrant	Non-penetrant	Penetrant	Penetrant
HIA	Highly absorbed	Moderately ab...	Highly absorbed	Highly absorbed	Highly absorbed	Highly absorbed	Highly absorbed
Metabolic Stability	Undefined	Undefined	Undefined	Undefined	Undefined	Undefined	Undefined
LogP	Optimal	Optimal	Optimal	Optimal	Lipophilic	Optimal	Optimal
MW	Good	Good	Good	Good	Good	Good	Good
H-Donors	Good	Good	Good	Good	Good	Good	Good
H-Acceptors	Good	Good	Good	Good	Good	Good	Good
Rot. Bonds	Good	Good	Good	Good	Good	Good	Good
Rings	Good	Good	Good	Good	Good	Good	Good
Lipinski	Good	Good	Good	Good	Good	Good	Good
Lead-like	Good	Good	Good	Good	Moderate	Good	Good
Solubility	Soluble	Soluble	Soluble	Soluble	Soluble	Soluble	Insoluble
P-gp Substrates	Non-substrate	Non-substrate	Non-substrate	Non-substrate	Undefined	Undefined	Undefined
Ames	Undefined	Non-mutagenic	Non-mutagenic	Non-mutagenic	Undefined	Non-mutagenic	Non-mutagenic
hERG	Non-inhibitor	Non-inhibitor	Non-inhibitor	Non-inhibitor	Non-inhibitor	Non-inhibitor	Undefined

4 ^{1}H NMR spectra for studied compounds 1-7

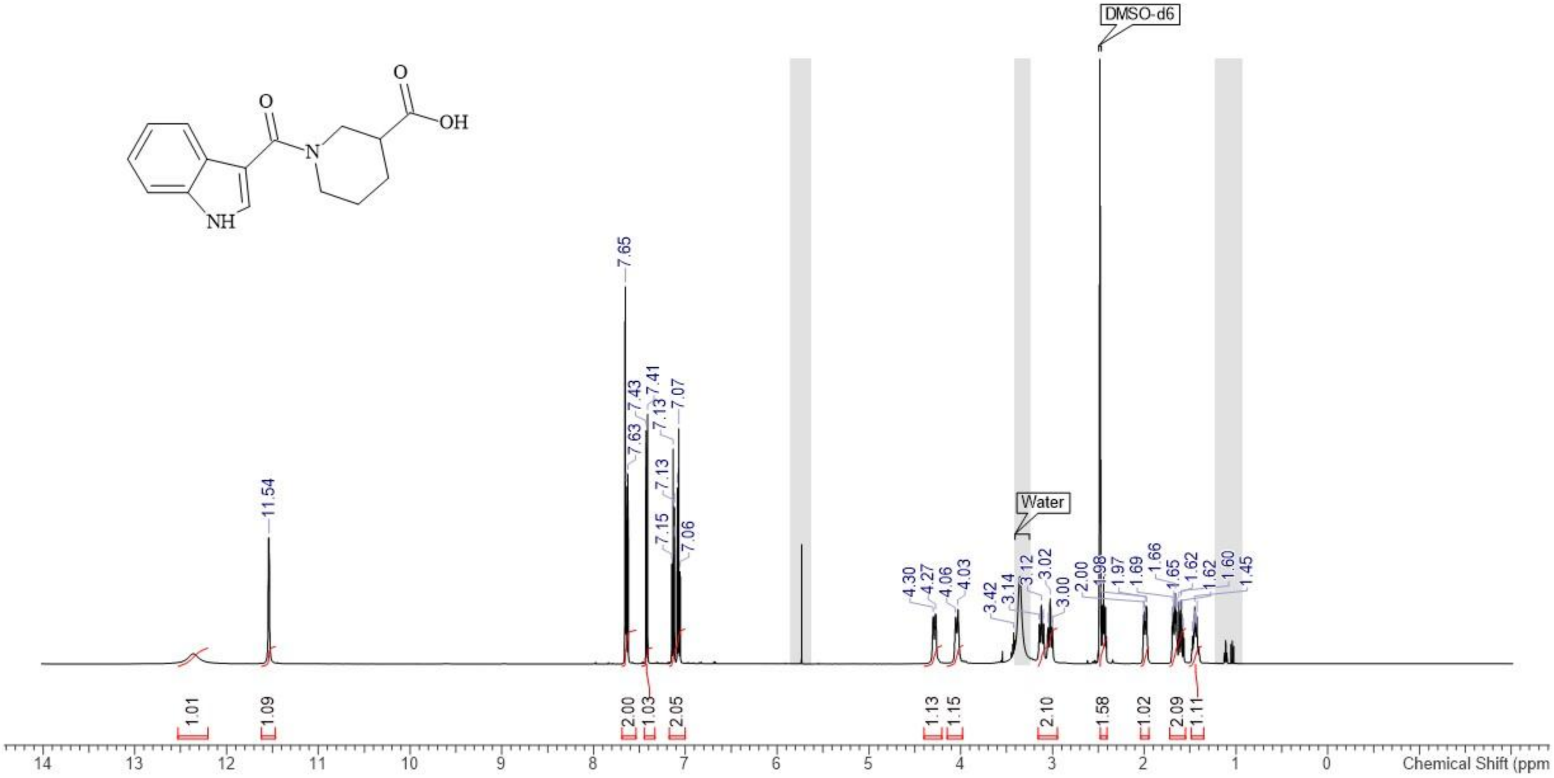

Figure S5: ^{1}H-NMR (500 MHz, DMSO-d_6) spectrum of 1-(1H-Indol-3-ylcarbonyl)-3-piperidinecarboxylic acid (1)

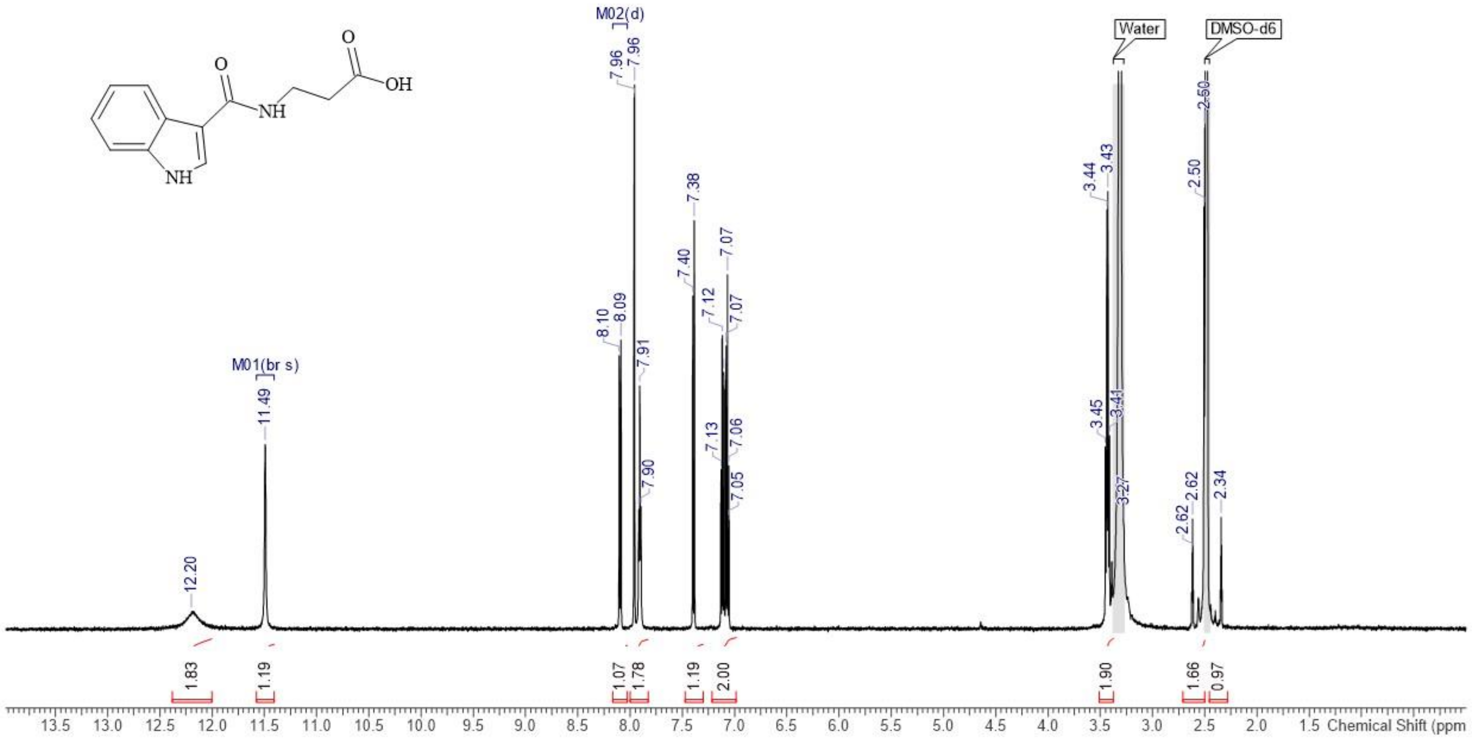

Figure S6: ^{1}H-NMR (500 MHz, DMSO-d_6) spectrum of 3-[(1H-Indol-3-yl)formamido]propanoic acid (2)

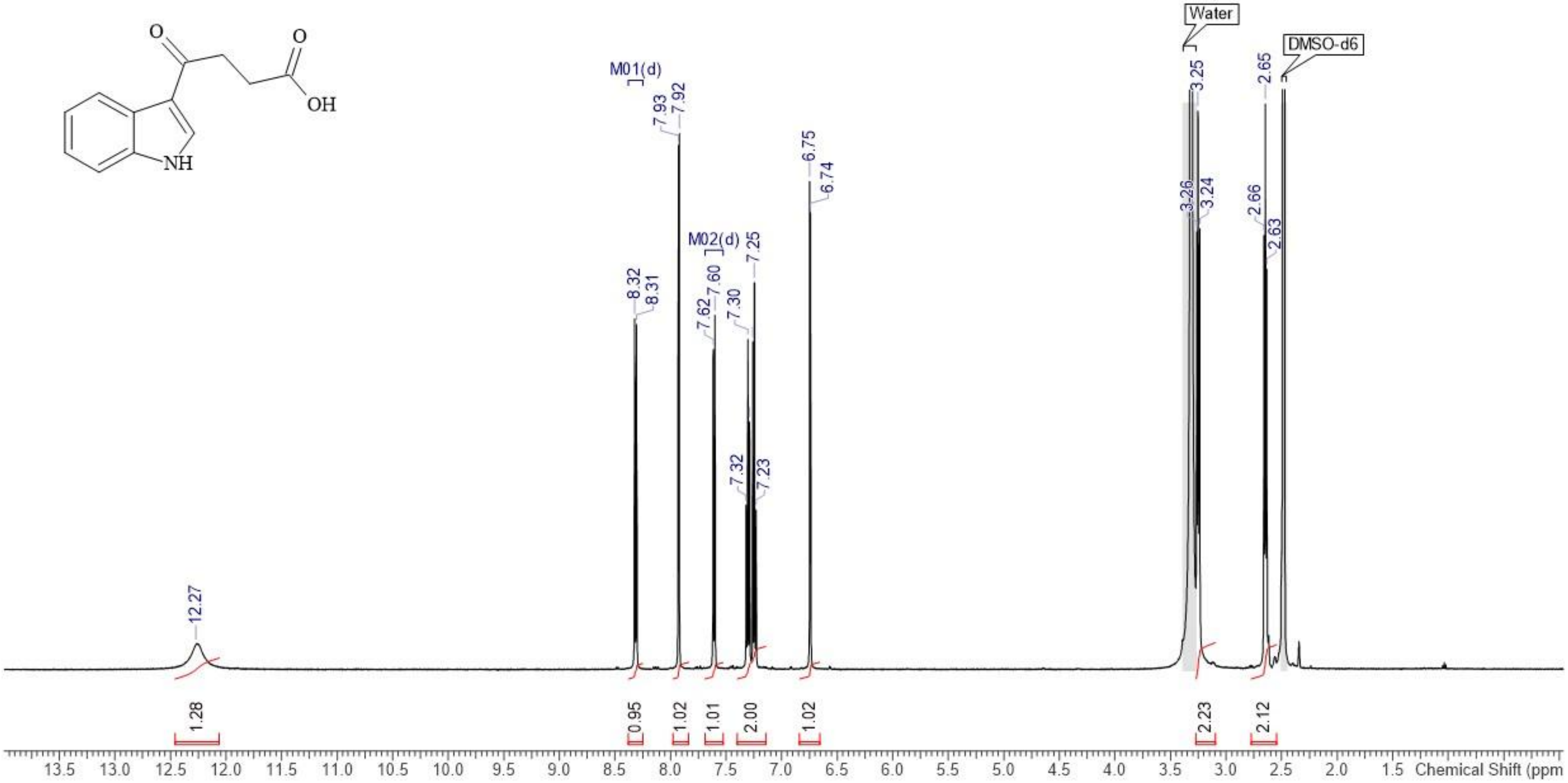

Figure S7: ^{1}H-NMR (500 MHz, DMSO-d_6) spectrum of γ-Oxo-1H-indole-3-butanoic acid (3)

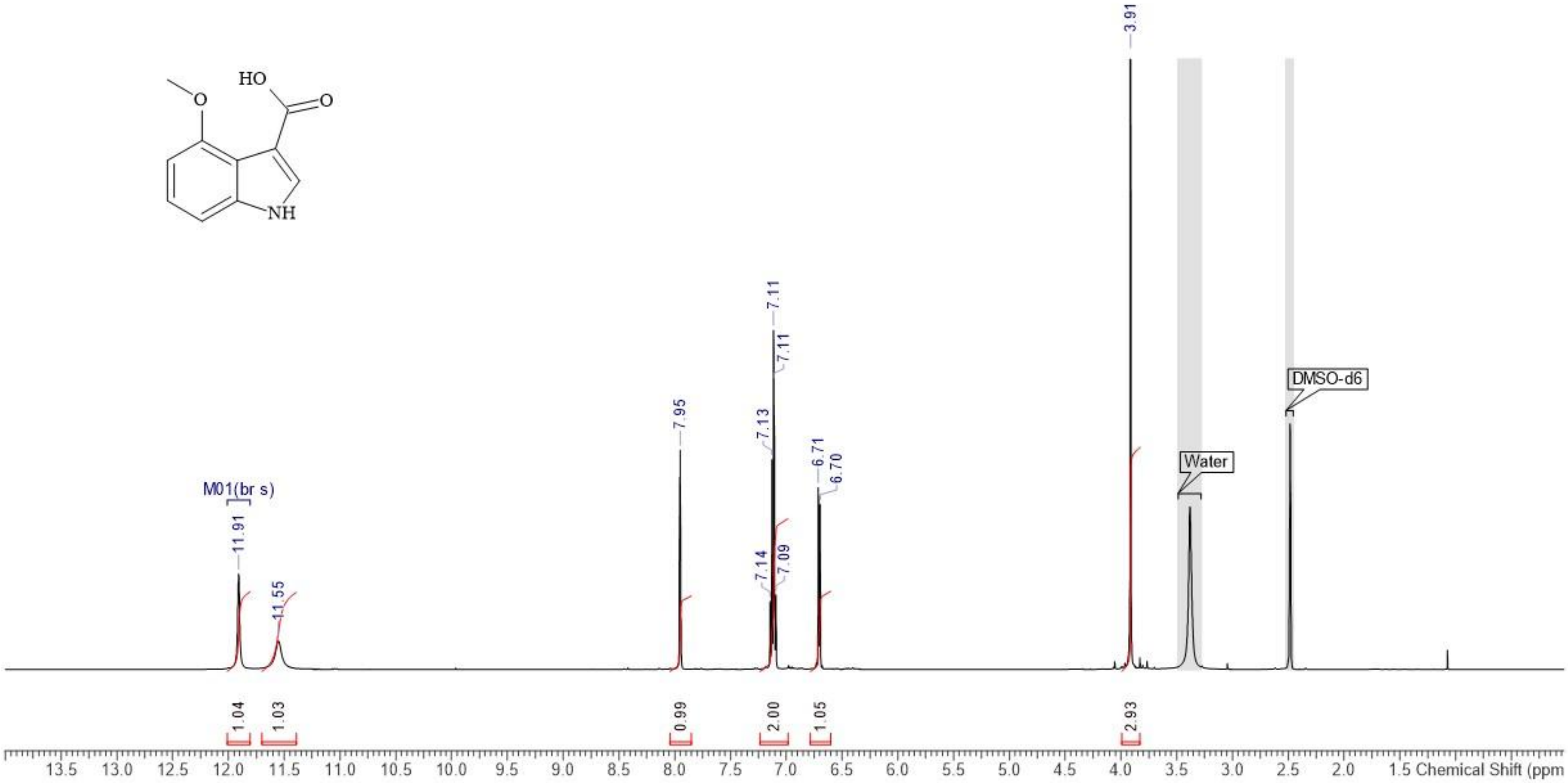

Figure S8: ^{1}H-NMR (500 MHz, DMSO-d_6) spectrum of 4-Methoxy-1H-indole-3-carboxylic acid (4)

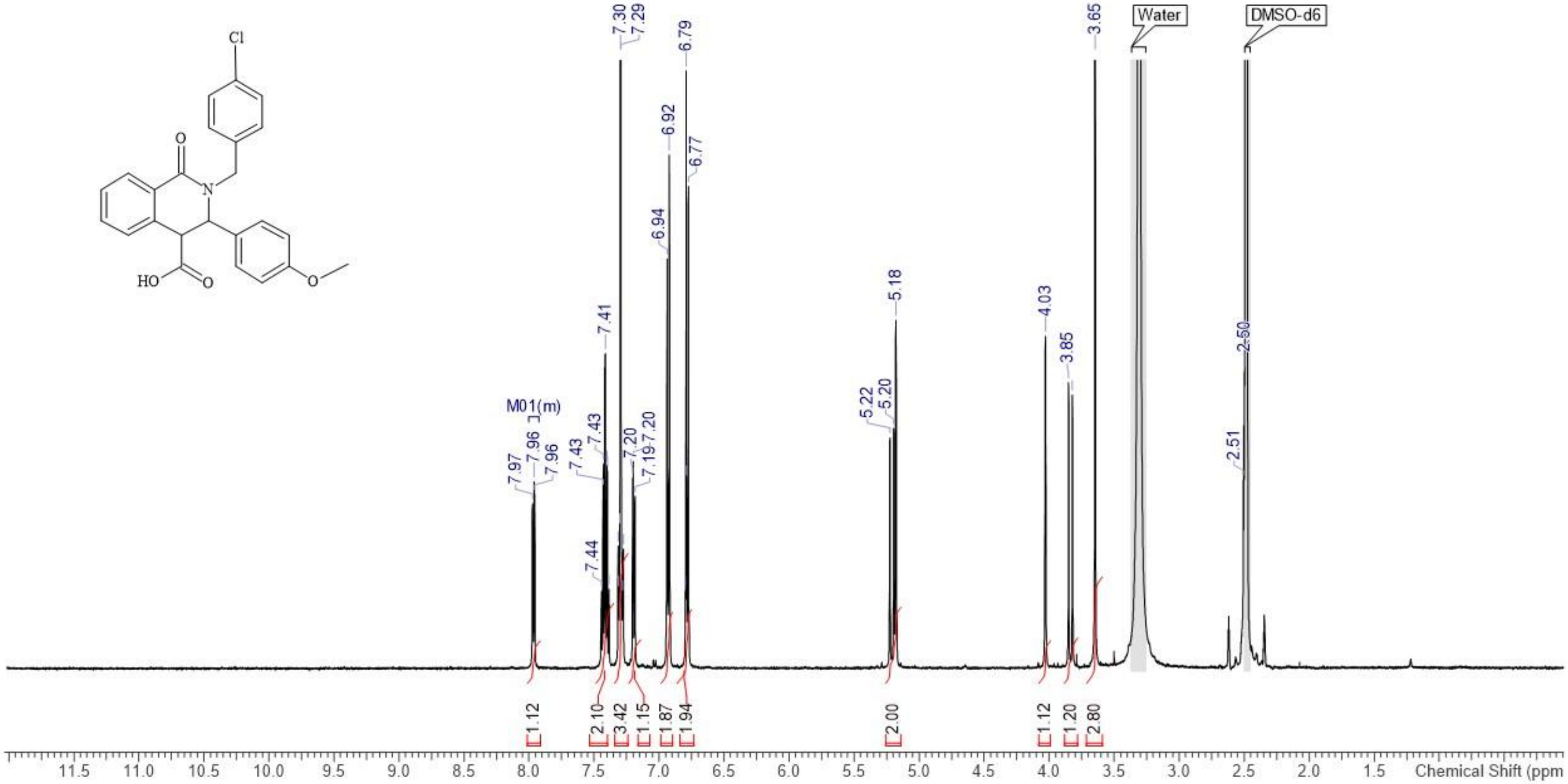

Figure S9: ^{1}H-NMR (500 MHz, DMSO-d_6) spectrum of (3R,4S)-2-[(4-Chlorophenyl)methyl]-1,2,3,4-tetrahydro-3-(4-methoxyphenyl)-1-oxo-4-isoquinolinecarboxylic acid (5)

Figure S10: ^{1}H-NMR (500 MHz, DMSO-d_6) spectrum of 3,4-Dihydro-6,7-dimethoxy-1-(1-methylethyl)-2(1H)-isoquinolinesulfonamide (6)

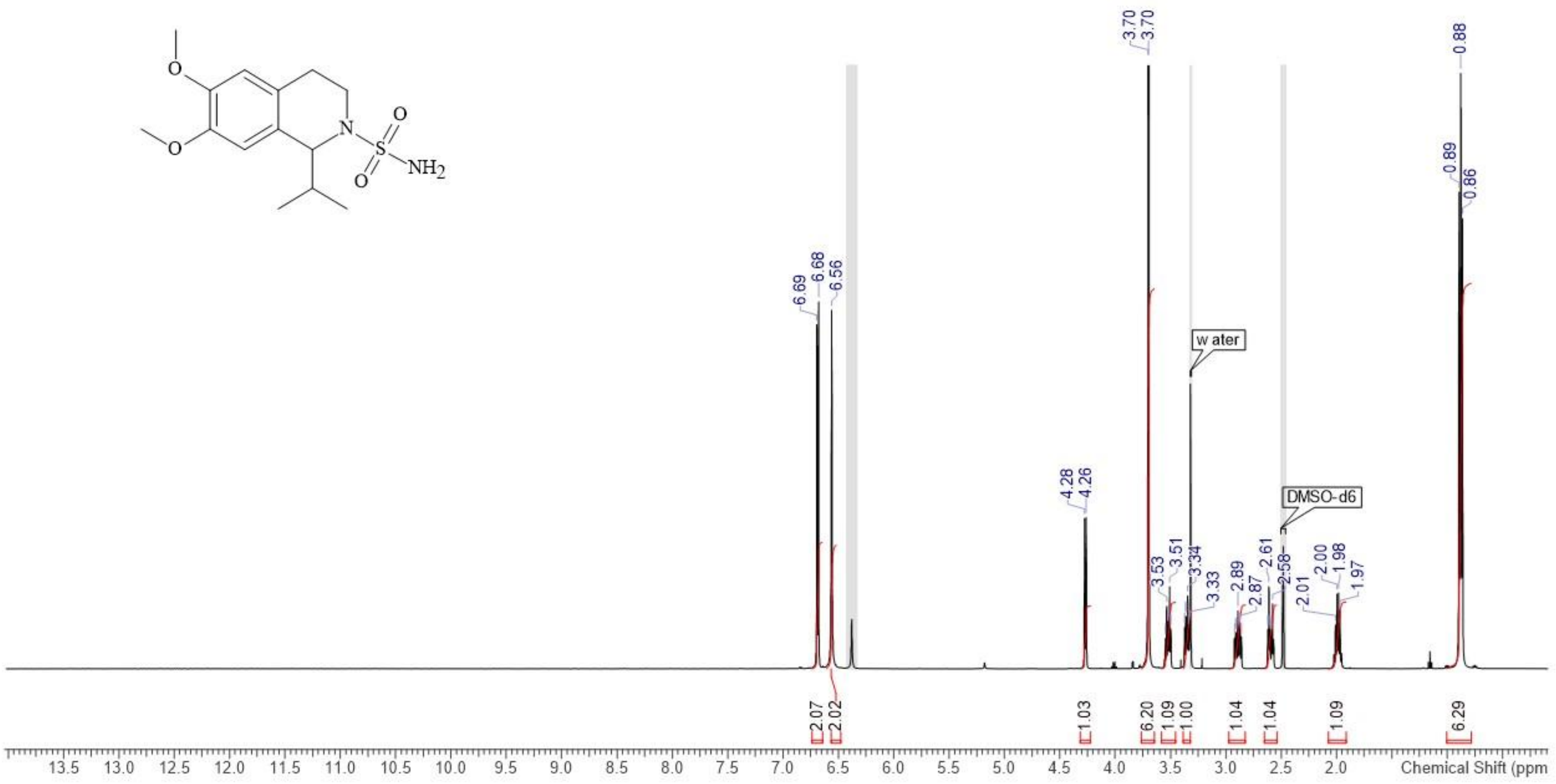

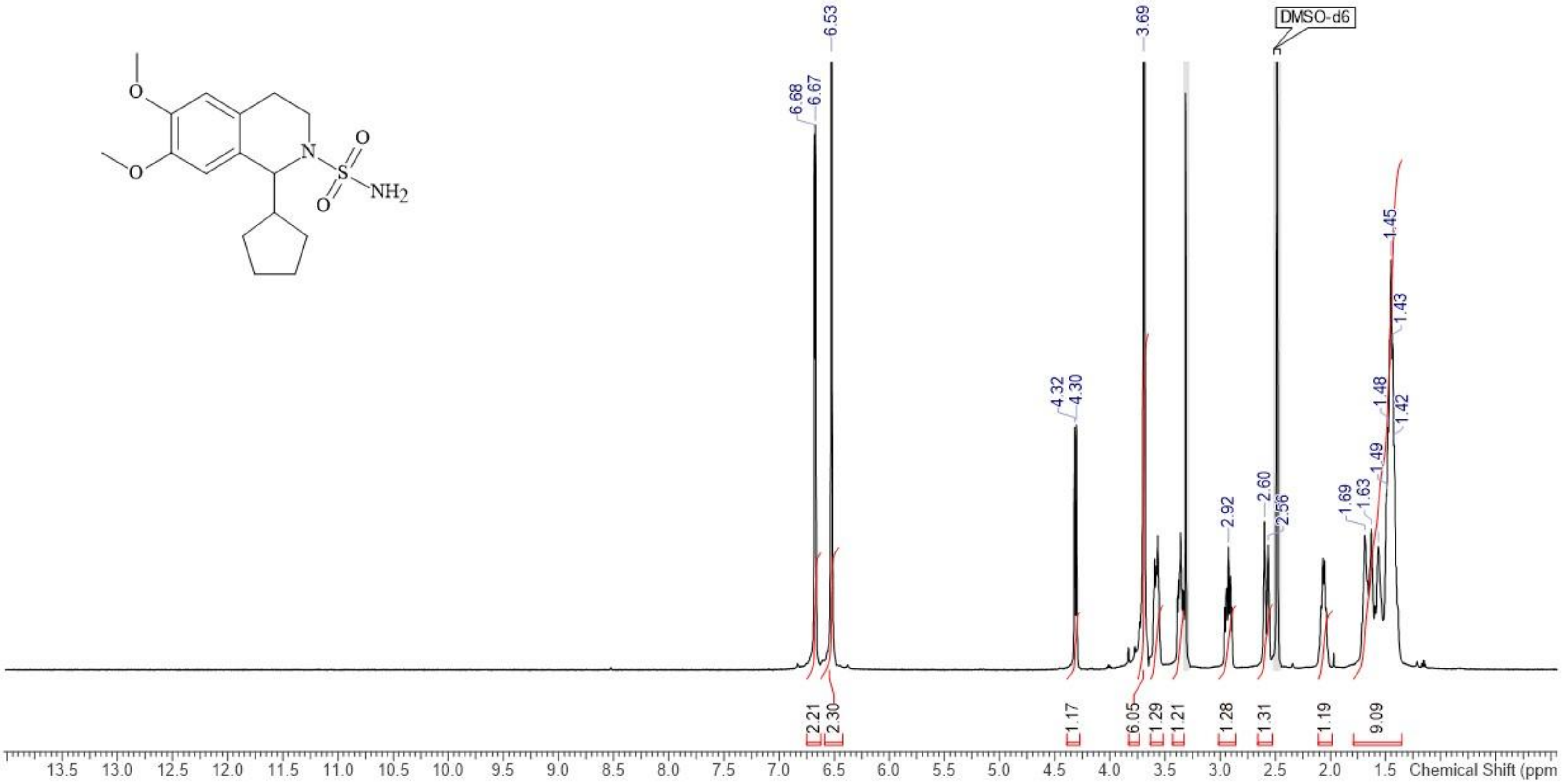

Figure S11: ^{1}H-NMR (500 MHz, DMSO-d_6) spectrum of 31-Cyclopentyl-3,4-dihydro-6,7-dimethoxy-2(1H)-isoquinolinesulfonamide (7)

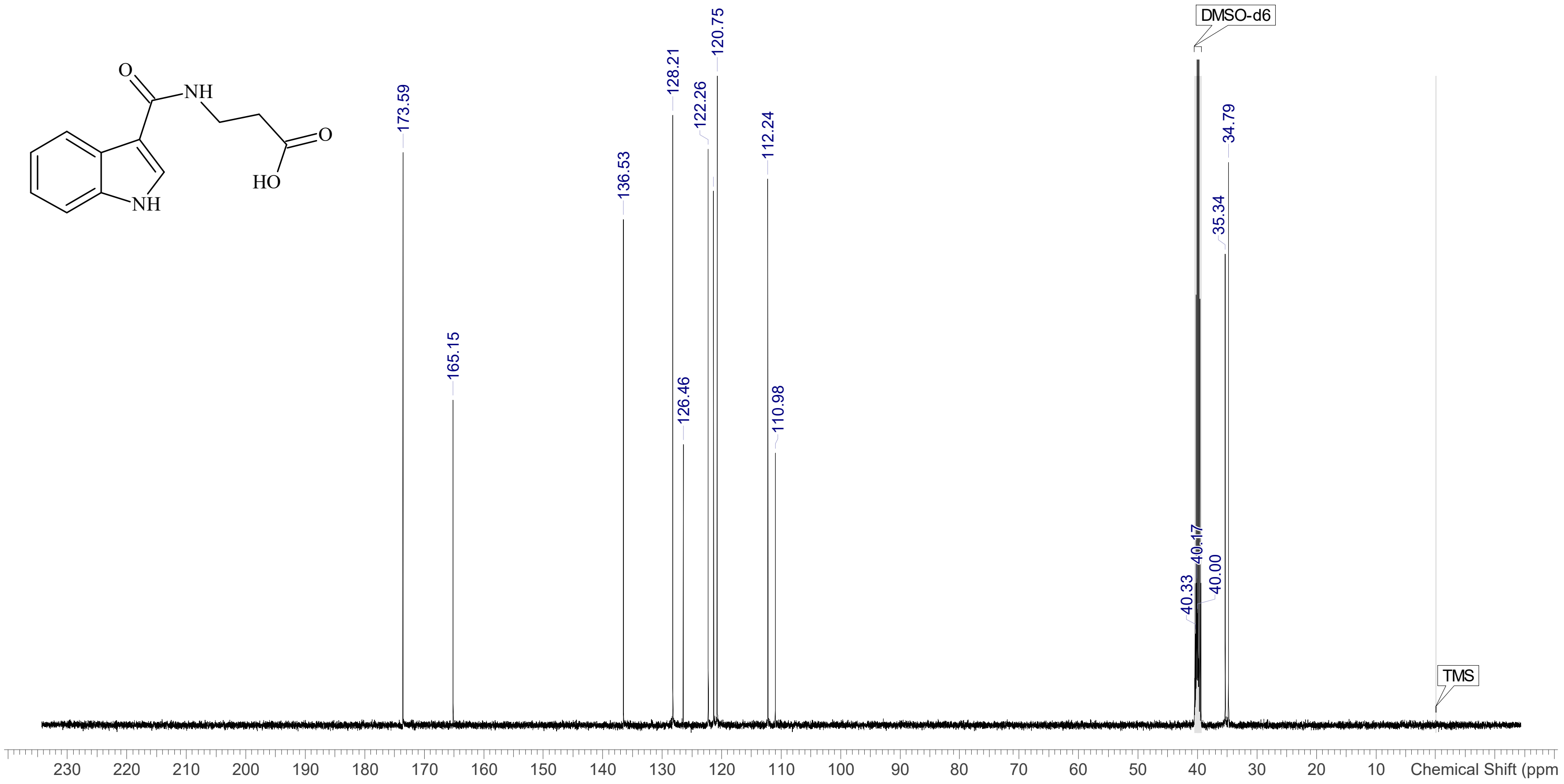

Figure S12: ^{13}C-NMR (126 MHz, DMSO-*d*6) spectrum of 3-[(1H-Indol-3-yl)formamido]propanoic acid (2)

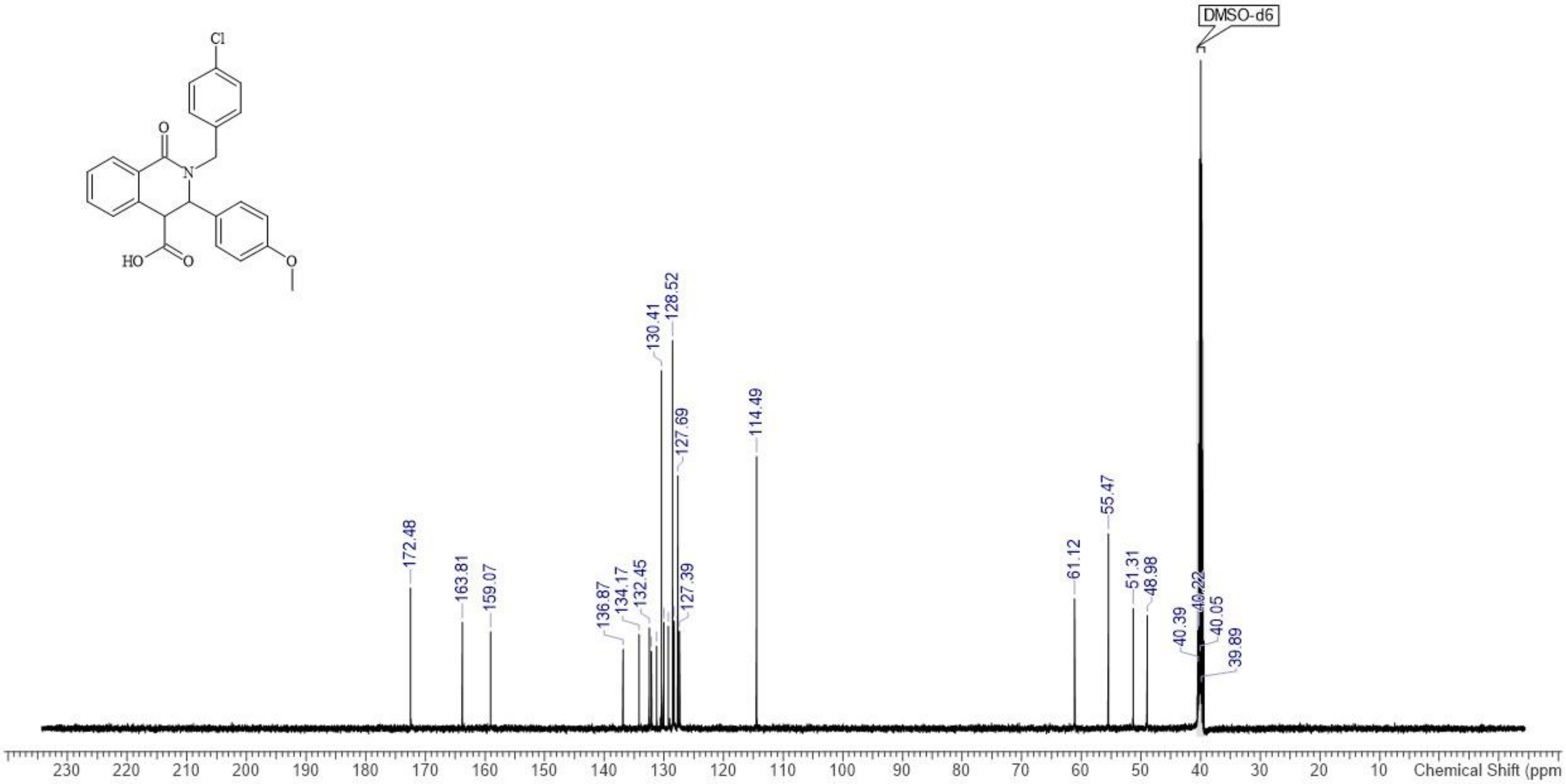

Figure S13: ^{13}C-NMR (126 MHz, DMSO-*d*$_6$) spectrum of (3R,4S)-2-[(4-Chlorophenyl)methyl]-1,2,3,4-tetrahydro-3-(4-methoxyphenyl)-1-oxo-4-isoquinolinecarboxylic acid (5)

5 Fluorescence interference controls for NA assay

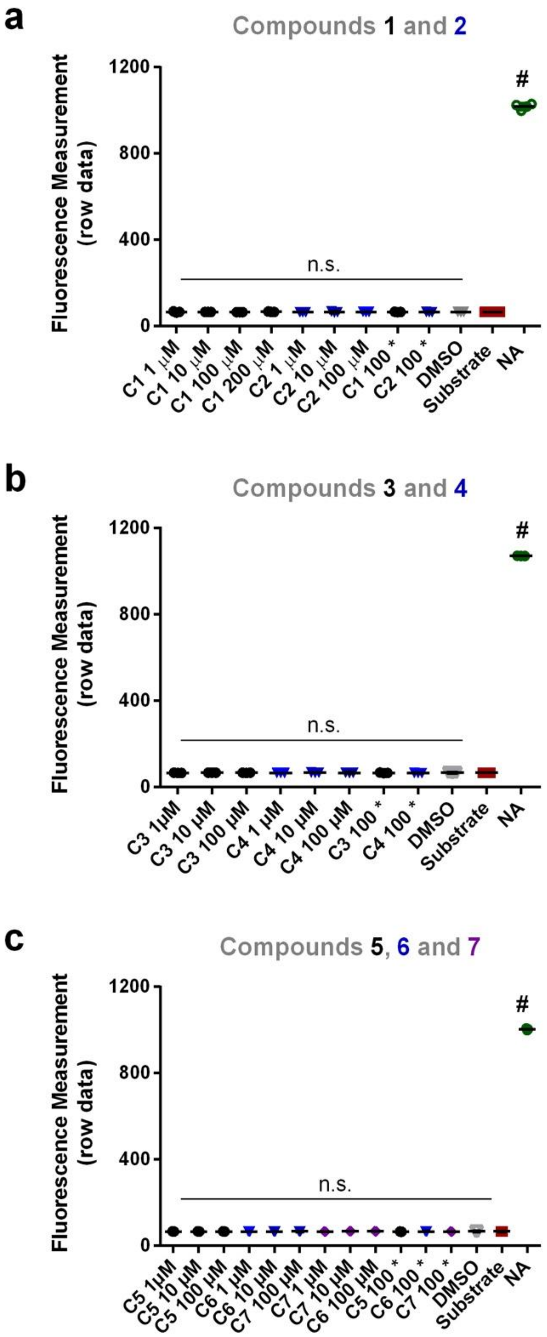

Figure S14. Raw fluorescence values obtained from "substrate + compound, no enzyme" controls for compounds **1–7** at 1, 10 and 100 µM. In the graphs, compound identifiers are shown as C1–C7 (i.e., compound 1 = C1, compound 2 = C2, etc.). For each compound, a sample containing the compound alone (no substrate) is indicated with "*". Panel (a) shows compounds **1** and **2**; compound **1** was additionally evaluated at 200 µM, and this value is included in the plot. Panel (b) shows compounds **3** and 4; panel (c) shows compounds **5, 6** and **7**. DMSO controls correspond to the highest solvent percentage present in each assay set (0.4%, 0.1% or 0.8%, respectively). Substrate alone (red symbols) and substrate + NA (green

symbols) are included for comparison. Data are presented as mean ± SD (n = 3). Statistical analysis was performed using one-way ANOVA followed by Bonferroni's post-hoc test: n.s. = not significant vs. substrate-alone control; # = $p < 0.001$ vs. substrate-alone control.

These controls, included in all NA inhibition assays, confirmed the absence of compound autofluorescence and the absence of fluorescence quenching of the MUNANA substrate.
